# Using discrete wavelet transform for optimizing COVID-19 new cases and deaths prediction worldwide with deep neural networks

**DOI:** 10.1371/journal.pone.0282621

**Published:** 2023-04-06

**Authors:** Erick Giovani Sperandio Nascimento, Júnia Ortiz, Adhvan Novais Furtado, Diego Frias

**Affiliations:** 1 Manufacturing and Technology Integrated Campus–SENAI CIMATEC, Salvador, Bahia, Brazil; 2 Department of Electrical & Electronic Engineering, Surrey Institute for People-Centred AI, Faculty of Engineering and Physical Sciences, University of Surrey, Guildford, United Kingdom; 3 State University of Bahia–UNEB, Salvador, Bahia, Brazil; Sejong University, REPUBLIC OF KOREA

## Abstract

This work aims to compare deep learning models designed to predict daily number of cases and deaths caused by COVID-19 for 183 countries, using a daily basis time series, in addition to a feature augmentation strategy based on Discrete Wavelet Transform (DWT). The following deep learning architectures were compared using two different feature sets with and without DWT: (1) a homogeneous architecture containing multiple LSTM (Long-Short Term Memory) layers and (2) a hybrid architecture combining multiple CNN (Convolutional Neural Network) layers and multiple LSTM layers. Therefore, four deep learning models were evaluated: (1) LSTM, (2) CNN + LSTM, (3) DWT + LSTM and (4) DWT + CNN + LSTM. Their performances were quantitatively assessed using the metrics: Mean Absolute Error (MAE), Normalized Mean Squared Error (NMSE), Pearson R, and Factor of 2. The models were designed to predict the daily evolution of the two main epidemic variables up to 30 days ahead. After a fine-tuning procedure for hyperparameters optimization of each model, the results show a statistically significant difference between the models’ performances both for the prediction of deaths and confirmed cases (p-value<0.001). Based on NMSE values, significant differences were observed between LSTM and CNN+LSTM, indicating that convolutional layers added to LSTM networks made the model more accurate. The use of wavelet coefficients as additional features (DWT+CNN+LSTM) achieved equivalent results to CNN+LSTM model, which demonstrates the potential of wavelets application for optimizing models, since this allows training with a smaller time series data.

## 1. Introduction

Facing crises such as the COVID-19 pandemic requires constant monitoring of the social and epidemiological variables that involve it. The COVID-19 pandemic has changed the daily lives of the entire world population, which has been threatened by an easily transmitted virus, the severe acute respiratory syndrome coronavirus 2 (SARS-CoV-2). Several strategies must be taken to hinder the virus proliferation, and these need to be based on correct and scientifically proven information, which can be greatly benefited by prior knowledge of possible scenarios to be observed in the future. The daily, weekly, or monthly contagion rate projection to the future helps the decision-making process from managers who need to formulate mitigation policies, which is important in the face of changes in the virus spreading behavior as seen with Delta and Omicron variants.

Since the pandemic began, several predictive modeling studies have been carried out to investigate and build the best tools to support the development of strategies to combat the crisis. The proposed approaches include epidemiological mathematical modeling, such as the SIRD model (Susceptible-Infected-Recovered-Deceased) [1]; autoregressive models, such as ARIMA (Autoregressive Integrated Moving Average) [2]; and deep learning models, such as recurrent neural networks of the type LSTM (Long-Short Term Memory) [3], GRUs (Gated Recurrent Units) [4], bidirectional and convolutional LSTMs [5], among others.

The literature review points out that deep learning models based on LSTM and CNN networks are the most effective and robust, allowing to make more accurate predictions in a greater number of countries and in a longer predictive horizon than the autoregressive and statistical methods [6, 7]. Arun Kumar et al. [6] did a comparative analysis of autoregressive and neural models to predict trends in COVID-19. They concluded that, by citing several works with similar results, the LSTM and GRU deep learning-based models outperformed the ARIMA and SARIMA statistical models for most countries.

However, the accuracy of these models, as well as the size of the forecast horizon, need to improve so that they can be adopted by public health agencies as a tool to support the management not only of the COVID-19 pandemic, but of future ones. In this sense, Jamishid et al. 2022 [7], after reviewing 80 papers on methods for estimating and predicting the spread of COVID-19 concluded not to recommend any of these reported prediction methods to be employed in WHO’s ongoing practice.

One of the main limitations of the pandemic forecast is the fact that it has a single input variable in the case of new daily cases, which can be two inputs in the case of daily deaths, if new cases are used as a supporting variable. That is, the number of features is extremely small. In the financial market, for example, several indicators can be used to predict the future price of an asset together with the price of the asset.

Therefore, in limited feature cases data augmentation techniques are applied to generate additional features that carry useful information to make a more effective prediction. Hazarika and Gupta [8] applied the wavelet transform for this purpose to predict numerical time series of cumulative COVID-19 cases using vector machines as a model. They showed an improvement in the accuracy of long-term forecasts (60 days). In this work we present a new approach to predicting COVID-19, which consists of using the wavelet transform to increase the number of input features of LSTM-based deep learning models built for this problem. This work aims to contribute to overcoming the technical gaps known so far, especially in two aspects: (1) the prediction horizon to achieve reasonable accuracy, which is generally less than one month; and (2) models trained and tested in a small number of countries.

To offer a model for long-term daily prediction (30 days) of new cases and deaths caused by COVID-19, this work compares different deep learning approaches. The models were trained and validated with available data from all countries in the world with COVID-19 registered cases, using as a basis the most suitable model for time series prediction with deep learning (the LSTM network) in four different configurations:

1. LSTM using the raw time series as features;
2. LSTM with initial one-dimensional CNN (1D-CNN) layers (hybrid model) using the raw time series as features;
3. LSTM using the time series generated after the wavelet transform using its coefficients as features; and
4. LSTM with initial 1D-CNN layers using the time series generated after the wavelet transform using its coefficients as features.

Therefore, this study offers the following contributions:

- The developed model uses data from 183 countries with COVID-19 cases registered in the database provided by the Center for Systems Science and Engineering (CSSE) at Johns Hopkins University [9], using the same deep learning architecture for training the models of confirmed cases and deaths. Thus, in addition to integrating information of all the diversity observed in the pandemic scenario among different countries, the study evaluates the possibility of using a single model that offers good predictive performance for any location in the world, both for the prediction of confirmed cases and deaths.
- The study compares four different configurations for the model, which allows assessing the contribution of the convolutional layers along with recurrent neural networks, besides the performance gain observed with the addition of the coefficients from Wavelet transform as input to the model.
- Wavelet implementation to be used in the pre-processing step as feature augmentation technique, in addition to minimizing the possible biases caused by Wavelet signals in predictive models, due to the use of future data for the decomposition.

This paper is organized into five sections, the first of which is the introduction. Related work on mathematical and statistical modelling, wavelet implementation and deep learning for forecasting time-series is presented in Section 2. In Section 3, we describe the methodology of the proposed approach for Wavelet implementation, the neural networks architecture and metrics used for performance evaluation. Section 4 presents numerical results and discussion on models’ performance, in addition to analyzing the predictions from the model for selected countries in the five inhabited continents of the world. Finally, our conclusions are presented in Section 5.

## 2. Related work

The potential of deep neural networks has been explored by numerous studies involving different areas related to the COVID-19 pandemic, such as for virus detection [10–12], classification of image exams such as X-Ray and computed tomography (CT) [13, 14], and time-series forecasting [15–17]. Considering the use of artificial intelligence on different battlefronts against the COVID-19 pandemic, El-Rashidy et al. [18] conducted a survey of the most important applications and found 5 types: diagnosis, transmission estimation, study of the characteristics of different populations and the effects of Covid-19, vaccine development and the study of other drugs, supporting applications.

With the objective to improve COVID-19 detection accuracy, Sedik et al. [11] performed a study on data-augmentation for improving CNN and ConvLSTM models. They presented an improvement on detection accuracy of 4% to 11%. Alakus and Turkoglu [10] found the LSTM deep learning model performs the best results for COVID-19 diagnosis from laboratory data. The authors argue that these models can help medical professionals validate data from laboratories and help to plan resource allocation, which is especially important in a context of limited resources, although they recognize that the sample size is insufficient, and the studies need to be deepened.

In line with time-series prediction proposals, Chakraborty and Ghosh [16] propose a hybrid model using stationary ARIMA and nonstationary Wavelet-based forecasting model to predict the occurrence of new cases of Covid-19 in five countries (Canada, France, India, South Korea, and the UK) for 10 days ahead. The authors also used an optimal regression tree algorithm to evaluate the risk of dying, considering some variables that help to explain greater risk depending on the country. The study points out that the approaches are useful for designing response policies, including lockdown measures and the increase in the number of hospital beds, in a context of few certainties regarding the behavior of a pandemic.

Classical ARIMA model approaches are also commonly used for this type of problem. Benvenuto et al. [19] carried out a study to predict the evolution of the COVID-19 pandemic, proposing an econometric model with data from January 20 to February 10, 2020. The objective was to implement an ARIMA model to predict the number of cases of COVID-19, considering its prevalence (number of existing cases) and its incidence (number of new cases). The authors considered their predictions adequate for the moment (very initial for the pandemic context), pointing out the following configurations as the most adequate for each analysis: ARIMA (1, 0, 4) for prevalence; ARIMA (1, 0, 3) for incidence. There is no clear analysis of the predictive capacity of the models.

Barría-Sandoval et al. [20] tested some models for predicting both confirmed diagnoses and deaths in Chile. They concluded that the ARIMA time series model was the most appropriate for the number of COVID-19 cases and the damped trend method is the best for predicting the number of deaths in Chile. However, the authors recognize that the dataset is limited, and other approaches may perform better in other contexts.

Khan and Gupta [21] use data from January 31 to March 25, 2020, and propose an ARIMA model (1, 0, 1) to predict the daily number of people infected in India for 50 days ahead. The authors went further and implemented a nonlinear autoregressive (NAR) neural network to compare the accuracy of the models. The performance tests were carried out with data from March 26 to April 4, 2020. ARIMA and NAR model performances were measured and compared using the value of the determination coefficient (R2), which revealed the approach with Neural Networks—the NAR model—as more appropriate, as it presented the highest R2 values of 0.97 (highest R2 values of ARIMA model was 0.95).

Rasjida, Setiawana and Effendib [22] predicted the number of Covid-19 cases and deaths for Indonesia with 5 months input data (March-July/2020). The researchers compare the predictions of the Savitzky Golay Smoothing and Long Short Term Memory Neural Network model (LSTM-NN). The LSTM-NN model had the best performance in the tests.

Aiming to develop a model that saves computational resources, Liu et al. [23] propose a hybrid modified Particle Swarm Optimization and Deep Neural Networks (PSO-DNN) to reduce the computational cost for choosing the optimal hyperparameters for DNN. To test the applicability of the modified PSO-DNN model, they demonstrate how social distancing worked for flattening the COVID-19 curve, considering the duration and mode of implementation in some cities of the United States.

The work of Ribeiro et al. [15] stands out by using a stacking-ensemble model for the forecast of the COVID-19 time series. They applied a stacking-ensemble model for short-term forecasting the cumulative number of confirmed cases one, three, and six days ahead, in ten Brazilian states with high COVID-19 incidence. They used four base models: (1) cubist regression (CUBIST), (2) random forest (RF), (3) ridge regression (RIDGE), and (4) support vector regression (SVR) with a Gaussian process (GP) as the metamodel. They also tested an ARIMA method, comparing forecasting performances of isolated and stacking-ensemble models using three common metrics. The authors found SVR to be the best model followed by the stacking model. The maximum forecasting error for six days ahead was 6.9%.

Among the main differences with this work, it can be highlighted: (1) the forecast horizon in this work is one to thirty days ahead, (2) the models are trained with data in the year 2020 and tested with data from January/2021 to March/2022, both for all countries in the world, (3) different deep learning models were evaluated, using LSTM, 1D-CNN and wavelet transform, and (4) the proposed model predicts the daily number of cases, while their study considers the cumulative number of cases, which configures two problems with different complexity. Given that the time series of cumulative cases vary more smoothly over time and have less noise than those of daily cases, the complexity of the problem in this work is greater. Furthermore, this work evaluates and applies the best wavelet transform to each time series (cases and deaths) using the method developed by Zucatelli et al. [24, 25], as described below.

After reviewing literature available for COVID-19 time series prediction, two aspects in the proposed models were identified as points to be improved: (1) The prediction horizon to achieve reasonable accuracy is from a few days to two weeks; and (2) The models were trained and tested in a small number of countries. On the other hand, two promising configuration modifications were not sufficiently explored: (1) the use of one-dimensional convolutional layers (1D-CNN) in the initial part of the models; and (2) the use of Discrete Wavelet Transform (DWT) to generate derived features aiming at increasing the learning abilities of the models.

The use of the wavelet transform linked to time series prediction models can increase the accuracy of the predictions, as found by Zucatelli et al. [24, 25] in a study of short-term prediction of wind speed in Bahia, Brazil. The study applied a discrete wavelet transform (DWT) to eliminate the noise components of the time series, concentrating the model in the most relevant set of data. After testing different topologies using multilayer perceptron (MLP) and recurrent neural network (RNN), the authors found that the hybrid algorithm composed of an RNN with wavelet decomposition using Meyer discrete function (dmey) obtained the best statistical result, which demonstrated that this is an adequate application.

Among the applications for the analysis of the COVID-19 pandemic behavior, Hazarika and Gupta [8] performed a modeling and prediction study of the disease spread, aiming at evaluating the predictive capacity of a random vector functional link (RVFL) network trained with Wavelet decomposed time-series data, provided as an input to the model. Data for the study came from the five most affected countries by the disease until July 2020: Brazil, India, Peru, Russia, and the USA. The prediction capacity for cumulative number of cases (60 days ahead) of the proposed WCRVFL network—a hybrid network using 1D discrete wavelet transform and random vector functional link (RVFL)—was analyzed in comparison with RVFL without wavelet and a support vector regression (SVR) model. The work of Hazarika and Gupta [8] demonstrated the high predictive performance of the hybrid wavelet-based approach and highlighted the potential of this model type in understanding the pandemic behavior over time. Guleria et al. [26] studied infection cure and death rate in regions of India comparing models from Decision Trees, Naïve Bayes, Support Vector Machines, and Ensembling methods. They proposed a hybrid model with a Fine-Tuned Ensemble Classifier which outperforms the conventional classifiers, even for unbalanced datasets. Most published predictive models of COVID-19 are trained with data from a single country, which brings the risk of overfitting due to the relative insufficiency of data in relation to the number of learnable parameters [27]. Overfitting implies that the model has memorized the training data by doing practically a perfect interpolation between them, but lacks the ability to interpolate new and different data. In the case under study, an overfitted model learned the behavior of the time series in the first epidemic waves in the country for which the deep learning model is built, and therefore there will be a risk of not being able to correctly predict future waves, mainly, if the pattern of formation of the new waves differs from that of the first waves used for training.

A natural way to avoid overfitting is to increase the diversity of the training set, preventing the model from making a perfect interpolation of all the waves it contains. To that end, we built a dataset of isolated epidemic waves with all waves observed in 183 countries around the world up to March 2022. It should be remarked, that because we build a dataset with data from all over the world, the built and trained model is capable of making predictions in any country, unlike other models that are developed for specific countries.

For the identification of epidemic waves and the division of time series into isolated waves, a novel method of noise filtering and identification of temporal cut-off points was developed. This method is briefly described in the Methodology section.

Additionally, as mentioned in the introduction, this is the first time that the wavelet transform is used to generate new features for the COVID-19 time series forecasting problem using LSTM deep learning model, in order to improve the accuracy and forecast horizon. Wavelets extract information from the spatial and frequency domains of time series of daily cases and deaths, presented in the form of numerical coefficients that are used as features. In this work, we chose the best wavelet family and the set of coefficients that most contribute to the improvement of the performance of our predictor built with deep learning models combining LSTM and CNN networks.

In Table 1 we list the analysis of two recent reviews and two selected papers with a detailed description of related works indicating the model architecture as well as the number of related papers matching each of the four features of the model described in this paper: (1) the use of wave-wise training data (creating training samples from time series describing single pandemic waves), (2) the use of wavelet data as complementary input features, (3) the development of a single predictor able to predict epidemic data (cases or deaths) in more than 100 countries, and (4) the ability to predict up to 30 days ahead.

**Table 1:** Analysis of related works focusing on features of the model described in this paper.

Review/paper author (year)	# of analyzed papers	model architecture			Wave-wise training data	Wavelet as features	single predictor for 100+ countries	30 days ahead prediction
		Neural Networks & Deep Learning	ARIMA-like & Statistical models	Other (including SIR-like models)				
Jamshidi et al. (2022) [7]	43	29	14	0	0	0	6	0
Comito C. et al. (2022) [28]	38	25	6	7	0	0	NA	2
ArunKumar et al. (2022) [6]	10	5	5	0	0	0	0	0
Chaurasia et al. (2020) [29]	5	0	5	0	0	0	0	0
TOTAL	96	59	30	7	0	0	6	2
This paper	1	Yes	No	No	Yes	Yes	Yes	Yes

It was found that none of the 96 relevant works analyzed used wavelet as complementary or main characteristics; that no work considered that the pattern to be learned was the formation and decay of isolated epidemic waves, as well as that only 2 models tried to predict 30 or more days ahead (the vast majority of them were developed to predict the value on the next day), as well as that only 6 models were trained with data from more than half of the countries in the world, aiming to build a single predictor for many countries.

## 3. Methodology

The problem addressed in this work was to build two deep learning predictors able to predict the future (from 1 to 30 days ahead) number of new daily cases and deaths in any selected country of the world having as input the time series of new cases and deaths in the selected country.

We built several models using multilayer LSTM network alone or combining 1D CNN with multilayer LSTM networks, to predict daily new cases and deaths from 1 to 30 days ahead. Each kind of model was built with and without auxiliary features created with wavelet transform of the input time series of new cases and deaths. Models differentiate each other in several senses: (1) number of previous days used (look-back window size), (2) wavelet family used for features augmentation and (3) set of wavelet coefficients selected as features.

The models were ranked according with the prediction error averaged for the whole test dataset containing epidemic waves of 183 countries.

### Data

Time series of daily new cases and deaths were retrieved from the COVID-19 Data Repository by the Center for Systems Science and Engineering (CSSE) at Johns Hopkins University [9]. The dataset contains the cumulative number of confirmed cases and deaths in 183 countries, being the most extensive and broad study of this nature. Data is preprocessed, filling eventual gaps and correcting negative values before converting into daily rates. The daily rate time series was then divided into single wave segments using a wave identification algorithm, which applies a two-parameter dependent non-shifting iterative smoothing technique, followed by a valley-peak-valley identification step. The day of the first confirmed case in each country is considered to be the first valley, while the last day of the series is considered a valley if it comes after a peak, or a peak if it comes after a valley. This procedure was implemented to deal with the multiwave nature of the COVID-19 pandemic behavior, from the perspective that the model must learn to predict the shape of epidemic waves, since in real-time applications the prediction will always have to be done in some stage of the evolution of the last epidemic wave, manifested in the data up to the current day.

**Fig 1:**
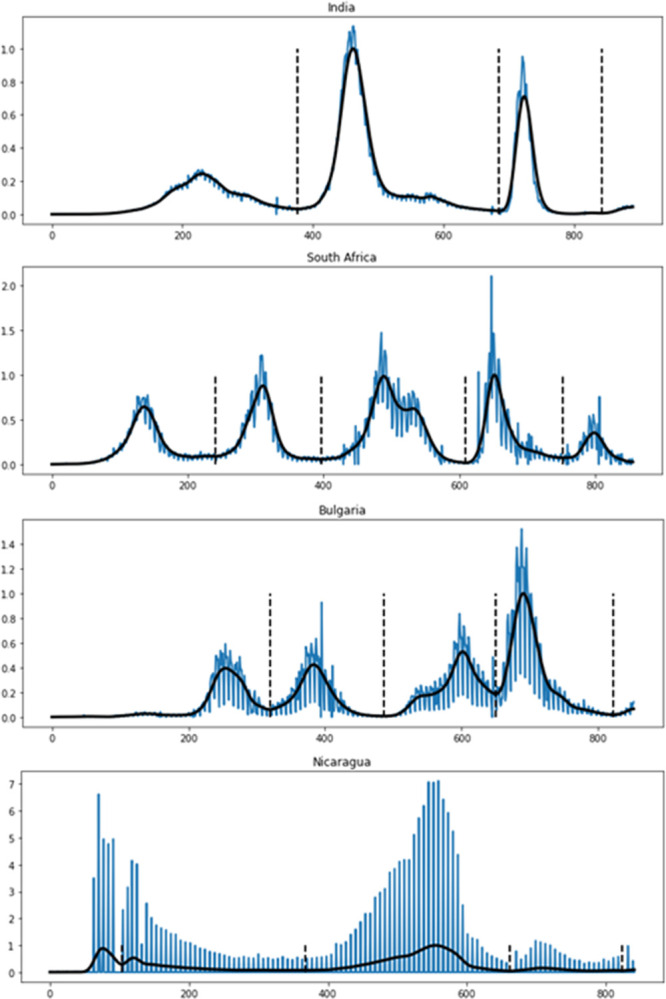
Blue line: Normalized time series of new daily confirmed cases in 4 countries with increased noise/signal ratio. Black curve: time series smoothed with the 3S method. Black dashed lines: wave limits.

Our model has been trained to be able to predict epidemic waves in such a way that, given the initial pattern of a rising wave, it can predict its maximum, and later, after the wave reaches its maximum, it can predict the wave’s decline. For this, instead of training with the entire time series that contains several waves, we divided the time series of each country into several subseries containing a single wave. To automate this division, we first need to filter the noise to find the valleys that delimit each wave, as shown in Fig 1.

Noise filtering was done using a symmetric moving window in order to avoid the shift of the smoothed time series that occurs with the usual non-symmetric windows. Furthermore, we used successive smoothing step with small window that proved to be more effective than single step smoothing with large windows. This novel noise filtering method was called Successive Symmetric Smoothing (3S).

Consider a symmetric window of size *w* = 2*h*+1, where *h* is the semi-width that will be used to smooth time series *Y*(*t*), *t* = 1,2…,*T*. Consider a succession of smoothed time series *y*_*n*_(*t*), *y*_*n*−1_(*t*),⋯,*y*_0_(*t*) such that *y*_*n*_(*t*) is obtained by smoothing *y*_*n*−1_(*t*), using the formula

$$y_{n}(t)=\frac{1}{1+min(T,t+h)-max(1,t-h)}\sum_{i=\max\left(1,t-h\right)}^{\min\left(T,t+h\right)}y_{n-1}(i),$$ (1)

n=1,2,…,N,t=1,2,…,T

where *y*_0_(*t*) = *Y*(*t*).

**Fig 2:**
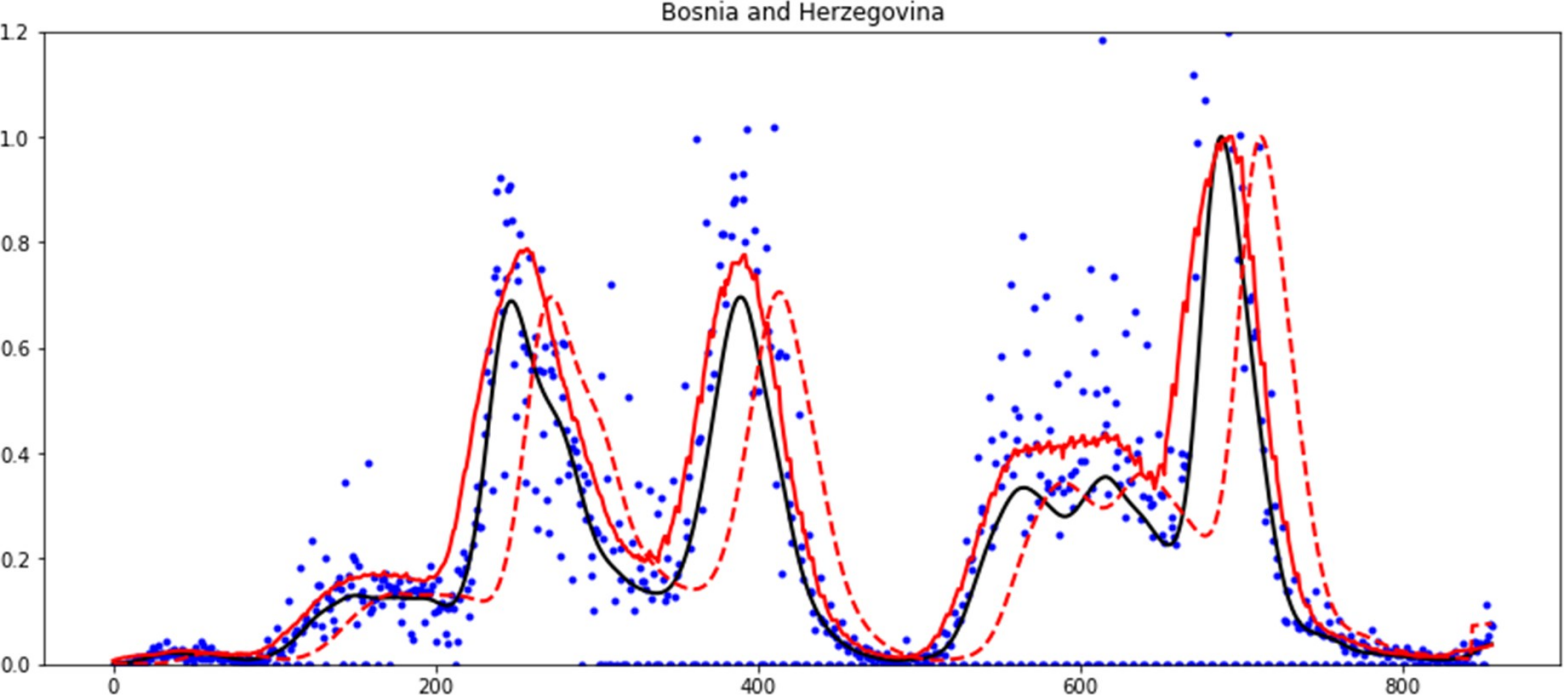
Blue dots: Time series of new cases in Bosnia and Herzegovina. Black line: the smoothed time series using 3S method with optimal parameters: window size w = 2*6+1 = 13 and four steps (n = 4). Red dashed line: the smoothed time series with 4 steps and “asymmetric” window of same size (w = 13). Red line: the smoothed time series with single step and symmetric window of equivalent size (w = 4*13).

The 2 parameters *h* and *n* were adjusted for the COVID-19 data. The parameter adjusting process was as follows: We look for valleys in the smoothed time series *y*_*n*_(*t*) knowing that there is a valley at time *t*_*v*_ if ${y_{n}(t_{v-1})\geq y}_{n}(t_{v})\leq y_{n}(t_{v-1})$. If the set of valleys found this way in a selected set of countries does not correspond with the expected by a human expert, it means that the parameters *h* and *n* need to be improved. This way we found *h* = 6 days and *n* = 4 as optimal values for all countries. The effectiveness was measured by the level of remaining noise and the flexibility of the smoothed curve. To illustrate the effect of the number of steps and window symmetry, in Fig 2 we show the time series of new cases in Bosnia and Herzegovina (blue dots) and with black line, the smoothed curve with the 3S method using window w = 2*6+1 = 13 and n = 4.

The curve with red dashed line is the smoothed series with the same number of steps and window size, but with the window not symmetrical. Note that it has the same shape as the one obtained with the 3S method but is shifted to the right. Finally, with a solid red line, we show the smoothed curve in a single step, using a symmetrical window of equivalent size, ie 4*13 days. It is observed that although there is no forward shift, there is residual noise and that in the region around the 600 days of the epidemic it is not able to detect the 2 small waves identified by the 3S method, which we call lack of flexibility of the smoothed curve.

Based on extensive experiments, it was found a look-back of 4 days to be optimal, comprising data of the current and the three previous days in the model input. The look-back is a hyperparameter that defines the size of the input window and has been optimized by varying it from 2 to 45 days. For each look-back value, the four models studied for each predicted variable (daily new cases and daily deaths) were trained with the complete dataset containing time series from 183 countries, from the beginning of the pandemic in December/2019 to December /2020. The performance of each trained model was then evaluated with the test dataset formed by the time series from January/2021 to March/2022, recording the performance metrics. Thus, it was found that 4 days is the optimal look-back for all models and for both predicted variables.

Analyzing the literature for this parameter, we found a recent article that makes a comparative analysis of autoregressive and neural models for predicting trends in COVID-19 [6]. In this article the look-back values chosen for 4 LSTM and CNN models are listed. Here, we reproduce these results in Table 2, showing the article, the model, the selected look-back and the countries for which the model was adjusted.

**Table 2:** Look-back values reported by authors working with LSTM and CNN models to predict COVID-19 time series.

Authors	Look-back (days)	Model	(number of countries) Countries
Nabi et al. 2021 [30]	3	CNN	Russia
Nabi et al. 2021 [30]	10	CNN	Brazil
Chandra et al. 2021 [31]	6	LSTM	India
Dairi et al. 2021 [32]	5	LSTM-CNN	(7) Brazil, France, India, Mexico, Russia, Saudi Arabia, US
**WEIGHTED AVERAGE**	5.4		

These results show that the optimal look-back, adjusted for time series from 7 different countries, varies slightly by country and by model, with a rounded average of 5 days. Therefore, the 4-day look-back found in our study with 183 countries is not outside the range found by other authors.

We built two models: one for new daily confirmed cases and one for daily deaths. Each of these models was trained with a set of time series resulting from dividing the time series of 183 countries into individual (unique) waves. In total, each dataset was composed of 737 single wave time series to train each model, which is equivalent to using 4 waves per country on average.

30 neurons were implemented in the output layers of the deep learning models to predict the number of cases and deaths in the next 30 days. The daily numbers of confirmed cases and deaths were used as basic features. In the architectures using wavelet transform, the wavelet coefficients obtained from both time series (see Wavelet application section below) were added to the feature set.

The deep learning models were trained and tested to forecast the daily number of confirmed cases and deaths, up to 30 days ahead. Time series were split into a training dataset containing data from the beginning of the pandemic until December 31, 2020, and a test dataset comprising data from January/2021 to March/2022.

### Wavelets application

Feature augmentation with discrete Wavelet transform was performed based on the method described in Zucatelli et al. [24, 25]. Time-series of the wavelet approximation (a) and detail (d) coefficients for each level were used as augmented features, which capture low and high-frequency information, respectively, in the decomposed time series.

Wavelets are square-integrable functions of a real variable *t* that describe different patterns of brief oscillations around *t* = 0. That is, as we move away from *t* = 0, *x*(*t*) can be expressed as a combination of base (orthonormal) scaling functions *φ*(*t*) and wavelets *ψ*(*t*) considering *S* scale levels, in the form

$$x_{S}(t)=\sum_{s=1}^{S}\left(\sum_{k}a_{s}(k)\varphi (\frac{t}{2^{s-1}}-k)+\sum_{k}d_{s}(k)\psi (\frac{t}{2^{s-1}}-k)\right)$$ (2)

where *a*_*s*_(*k*) are the approximate coefficients resulting from a bank of low-pass filter bank at scale *s*, and *d*_*s*_(*k*) are the coefficients of detail resulting from a bank of high-pass filter bank at scale *s*.

The approximate and detailed coefficients are obtained as the inner products of the time series with the scale and wavelet functions of the level *s*, respectively, shifted by *k* time steps, that is,

$$a_{s}(k)=\left\langle x(t),\varphi (\frac{t}{2^{s-1}}-k)\right\rangle$$ (3)

and

$$d_{s}(k)=\left\langle x(t),\psi (\frac{t}{2^{s-1}}-k)\right\rangle$$ (4)

Choosing a wavelet family, the scale and wavelet functions are defined. Note that equation *x*_*S*_(*t*) represents the decomposition of the input time series *x*(*t*) into 2*S* additive time series, each of which can be used as a complementary feature. More precisely, we have *S* time series given by

$$y_{a,s}(t)=\sum_{k}a_{s}(k)\varphi (\frac{t}{2^{s-1}}-k),\;s=1,2,\ldots ,S$$ (5)

plus *S* time series given by

$$y_{d,s}(t)=\sum_{k}d_{s}(k)\psi (\frac{t}{2^{s-1}}-k),\;s=1,2,\ldots ,S$$ (6)

such that

$$x_{S}(t)=\sum_{s=1}^{S}y_{a,s}(t)+y_{d,s}(t)$$ (7)

In the feature engineering step of the model building process, you need to choose: (1) the wavelet family, (2) the maximum significant level *S* and (3) the subseries to be used as complementary features.

Training and testing datasets went through a pre-processing step to select the best Wavelet transform family for processing the daily case and death signals. The best family selection to be applied to each of the variables was performed based on the analysis of the values found from the RMSE averages calculation by each one of them, considering the application of the first level for all families. After selecting the best Wavelet families to be implemented in each time series (cases and deaths), the performance of the models with DWT was evaluated for each transform level, achieving the best results with the insertion of levels 1 to 4.

Thus, as an increment to the input data for the models, the approximation and detail signals of the Wavelets transform were inserted in four levels for the series of confirmed cases and deaths. The input variables for each trained network are presented in Table 3 below.

**Table 3:** Input variables for each model.

Model	Raw data	Wavelet transform signals			
		Cases		Deaths	
		Approximation	Detail	Approximation	Detail
With Wavelet transform	date; country/region; number of cases; number of deaths.	Level 1	Level 1	Level 1	Level 1
		Level 2	Level 2	Level 2	Level 2
		Level 3	Level 3	Level 3	Level 3
		Level 4	Level 4	Level 4	Level 4
Without Wavelet transform		-	-	-	-

In order to avoid biases in the models’ prediction by obtaining information captured from future data in the Wavelets signals decomposition, the implementation of the four levels of Wavelets was carried out considering 183 time series (one per country) of six months starting in March/2020 and adding one day at a time for generating the coefficients. After this implementation, only data related to the series from September/2020 to December/2020 (daily number of cases, daily number of deaths, and Wavelet signals) were separated for training the models using Wavelets. Models without using Wavelets were trained with the complete 2020 series (March/2020 to December/2020) for 183 countries.

### Deep learning models

Two deep learning architectures were tested, one combining three 60 neurons LSTM layers with a Dropout and 30 neurons Dense layer, and another combining two 40 filters 1D-CNN layers, three 60 neurons LSTM layers with a 1D-Max-Pooling and a 30 neurons Dense layer. Models were trained in a supercomputing environment and developed in Python language using Keras, from the TensorFlow library. Adam optimization was used for the LSTM training. The CNN+LSTM network was trained using the AdaMax optimizer. Both are used as stochastic optimization methods and they are appropriate in this case because of the large amount of data. In order to improve the models, a fine-tuning procedure was undertaken to select optimal hyperparameters, using a randomized optimization by implementing RandomizedSearchCV from scikit-learn.

The configurations of the models are described in Tables 4 and 5.

**Table 4:** LSTM architecture.

Layer	Type	Neurons	Activation
Input	LSTM	60	-
1^st^ hidden	LSTM	60	TanH
2^st^ hidden	Dropout (0.1)	-	-
3^st^ hidden	LSTM	60	TanH
Output	Dense	30	ReLU

**Table 5:** CNN+LSTM architecture.

Layer	Type	Filters	Size	Padding	Neurons	Activation
Input	1D-Conv	40	2	SAME	-	TanH
1^st^ hidden	1D-Conv	40	2	SAME	-	TanH
2^st^ hidden	1D-Max-Pooling	-	2	SAME	-	-
3^st^ hidden	LSTM	-	-	-	60	TanH
4^st^ hidden	Dropout (0.1)	-	-	-	-	-
5^st^ hidden	LSTM	-	-	-	60	TanH
6^st^ hidden	LSTM	-	-	-	60	ReLU
Output	Dense	-	-	-	30	ReLU

LSTM models were trained for 300 epochs, with a batch size of 100, and a learning rate of 1e-4. CNN+LSTM models were trained for 182 epochs, with a batch size of 1000, and a learning rate of 0.001.

### Performance evaluation

The prediction performance of the models was evaluated using four different metrics: MAE, NMSE, Pearson R, and Factor of 2. Each metric measures in a different way the (dis)agreement between the predicted and real values. The Mean Absolute Error (MAE) and the Factor of 2 are very sensitive to shift from the mean. The Normalized Mean Squared Error (NMSE) measures the dispersion of the real data with respect to the predicted values. The Pearson’s R measures the linear correlation between real and predicted data which reflects how coherent the trend in the real data and the predicted trend are. A well-performed model minimizes MAE and NMSE, and maximizes Pearson R and Factor of 2.

## 4. Results and discussion

**Fig 3:**
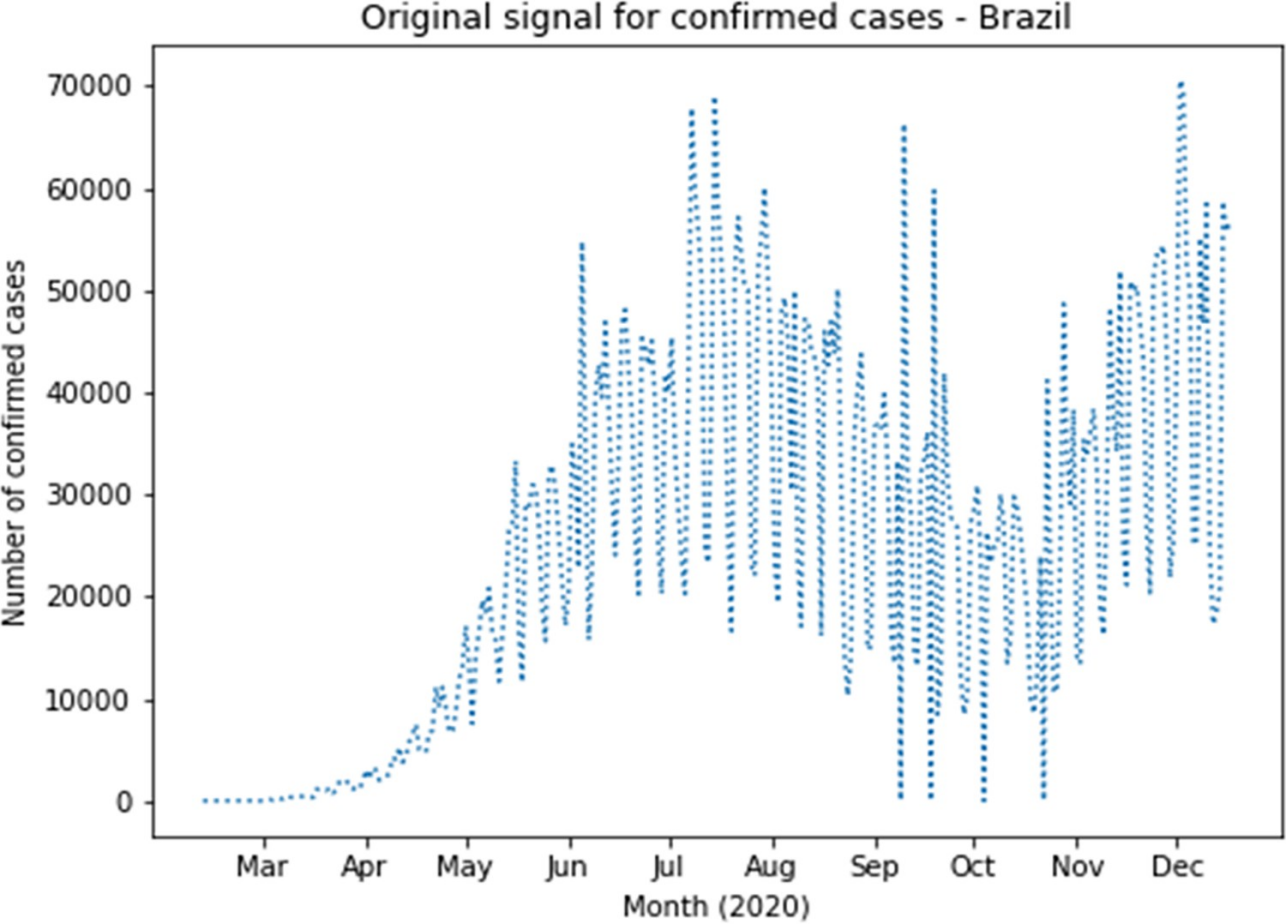
Daily confirmed cases in Brazil.

**Fig 4:**
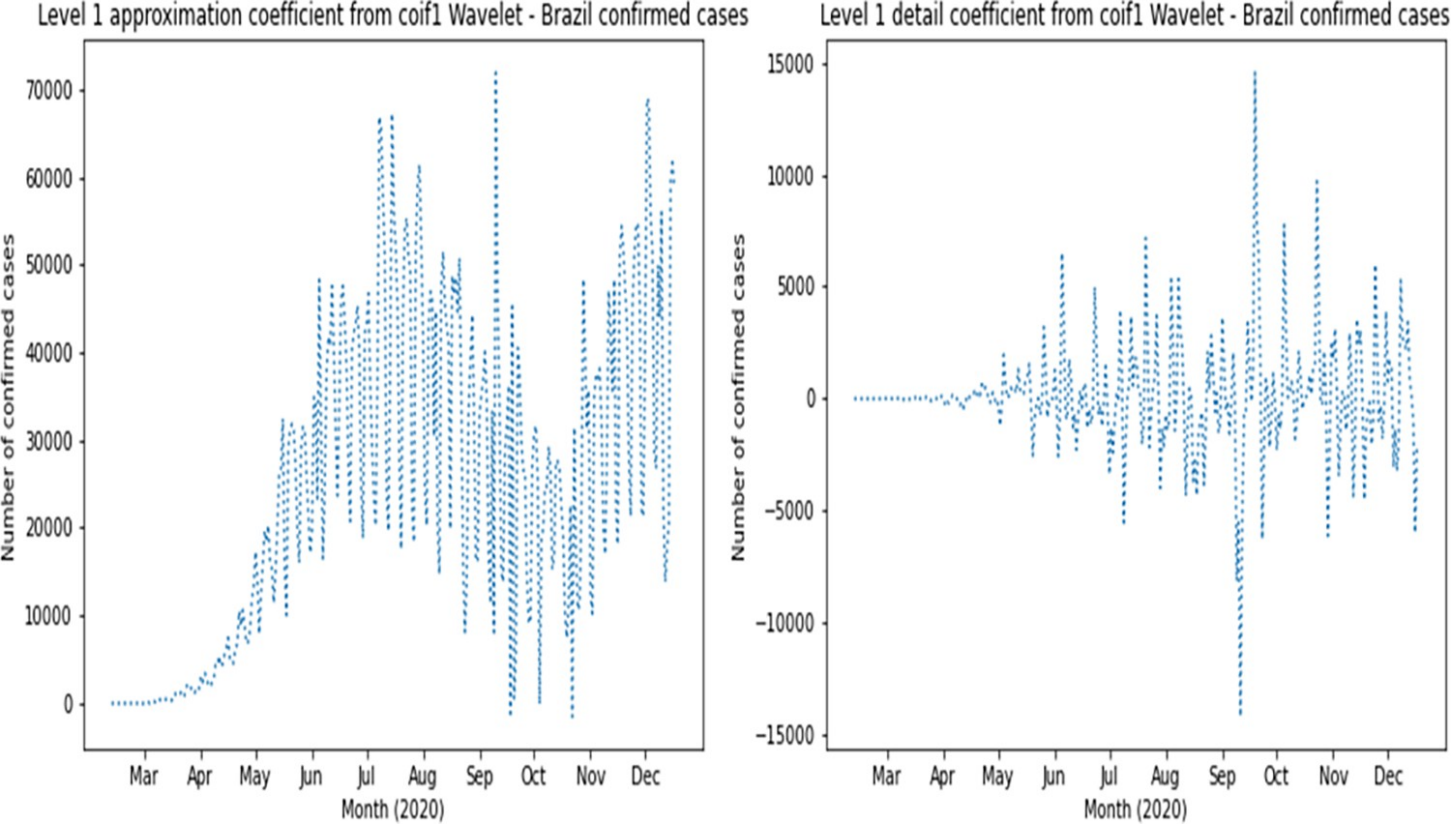
Wavelet transform signals for daily confirmed cases in Brazil.

**Fig 5:**
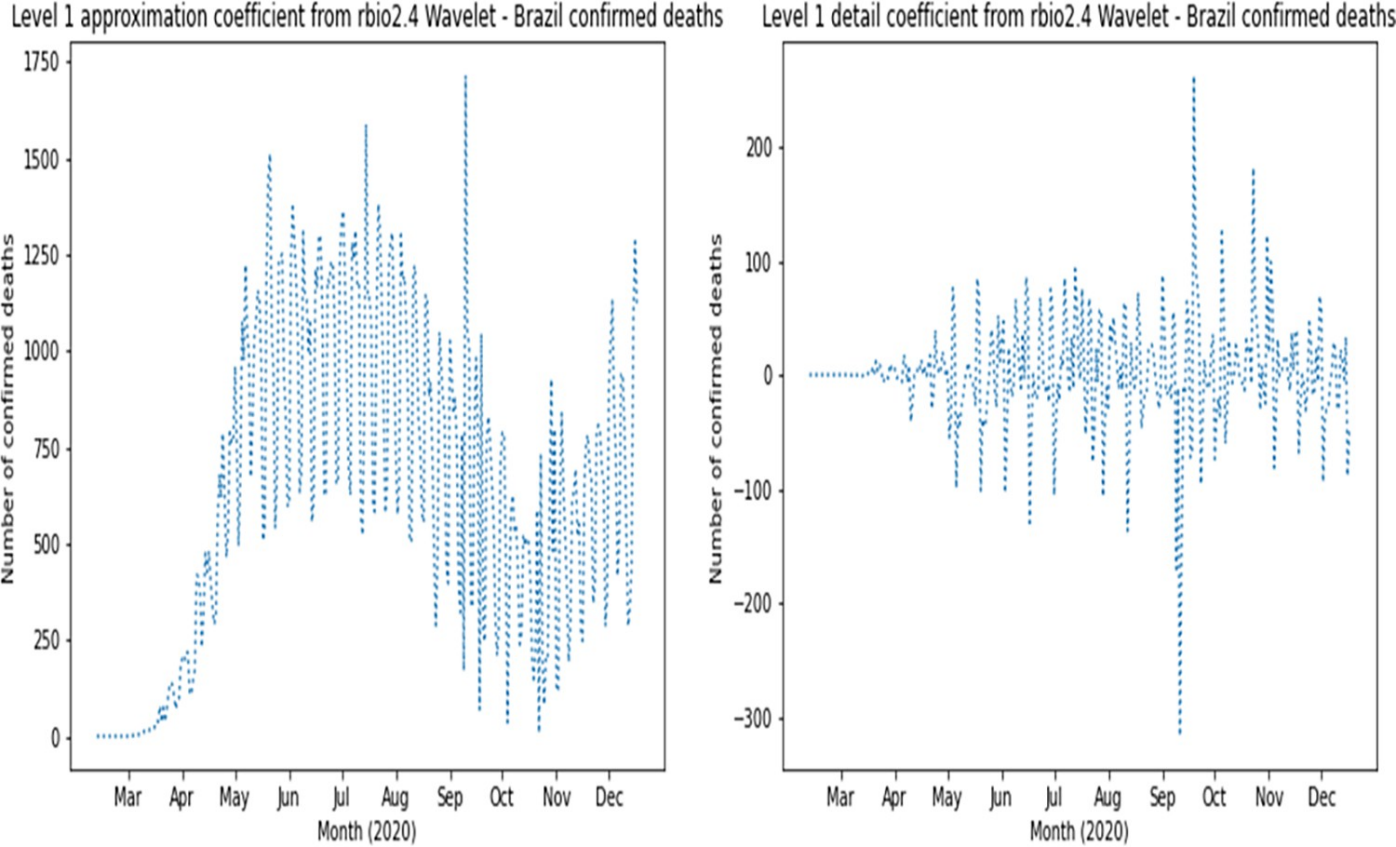
Wavelet transform signals for daily confirmed deaths in Brazil.

**Fig 6:**
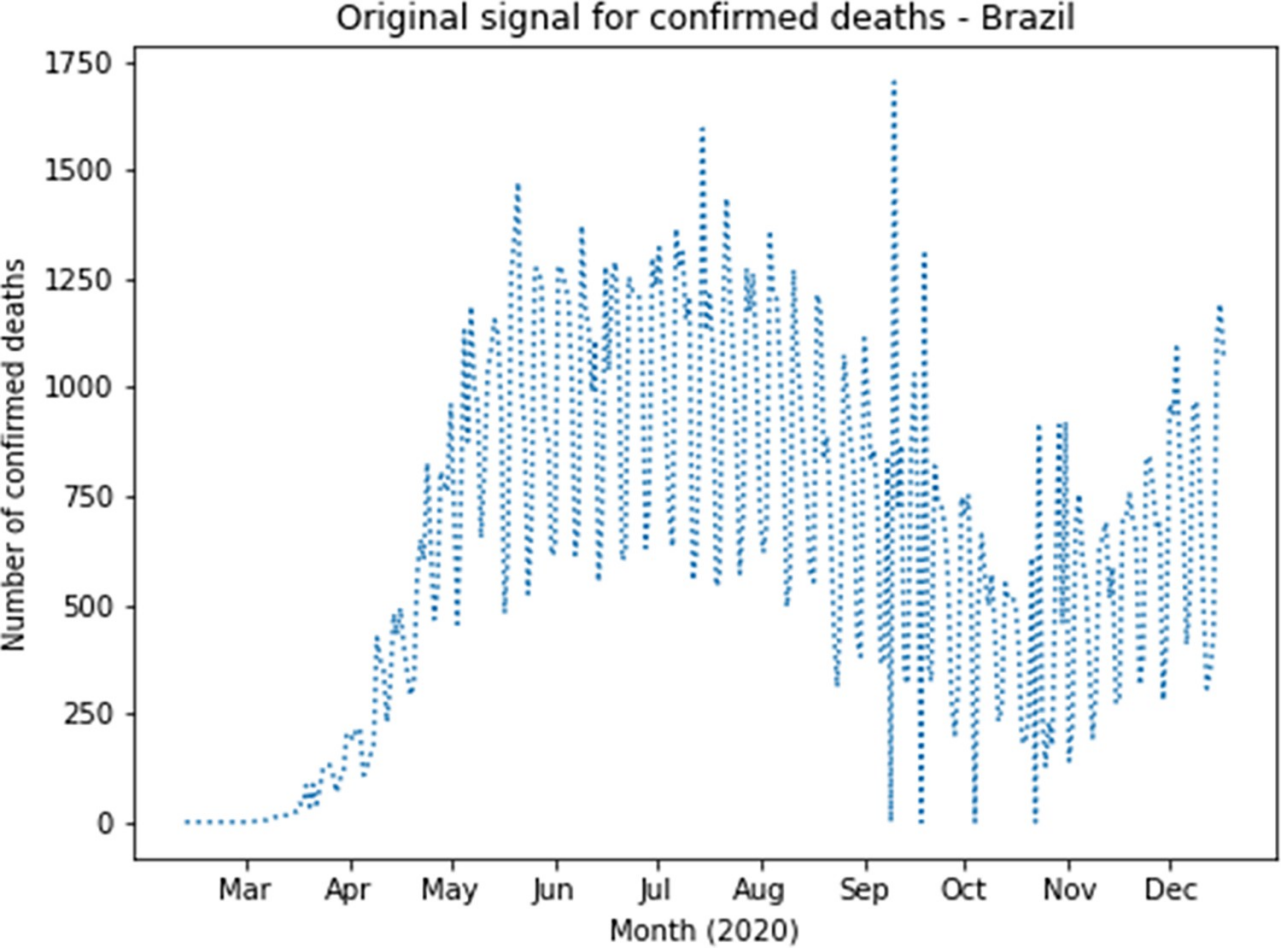
Daily confirmed deaths in Brazil.

The complete dataset was augmented with reconstructed approximation and detail signals of the first four Wavelet levels for confirmed cases and deaths. Based on RMSE results, the following families had the best values for confirmed cases: coif1, rbio2.4, rbio4.4, bior2.4 and bior2.6. The following families had the best values for deaths: rbio2.4, coif2, sym4, rbio6.8 and rbio2.6. Therefore, coif1 Wavelet was used for confirmed cases and rbio2.4 Wavelet was used for deaths. As an example, Fig 3 presents the original curve of confirmed cases in Brazil, and the effects of noise elimination in each country time series can be verified as shown in Fig 4, which presents the first level application of approximation and detailing signal of coif1 Wavelet to the original curve of confirmed cases in Brazil. This can also be seen in the signals corresponding to level 1 of rbio2.4 Wavelet presented in Fig 5, which smooth the original series of the daily number of confirmed deaths, also in Brazil (Fig 6). Wavelet coefficients were applied for 183 countries in this study, Brazil is used here as an example.

The models for confirmed cases and deaths were trained by adding four level Wavelet signals of approximation and detail coefficient applied to the two variables, which resulted in inserting more 16 input variables, in addition to the actual daily values of confirmed cases and deaths. After performing the training step, files with the models’ weights were generated, which were then used to make the inference for the 2021 dataset. The inference was performed with 30 time-steps predictions for cases and deaths values. Predictive ability was analyzed from metrics comparing predicted values to actual values in the dataset. Furthermore, the models were also compared to their versions without the Wavelets signals, i.e. using only the raw time series of confirmed cases and deaths.

**Fig 7:**
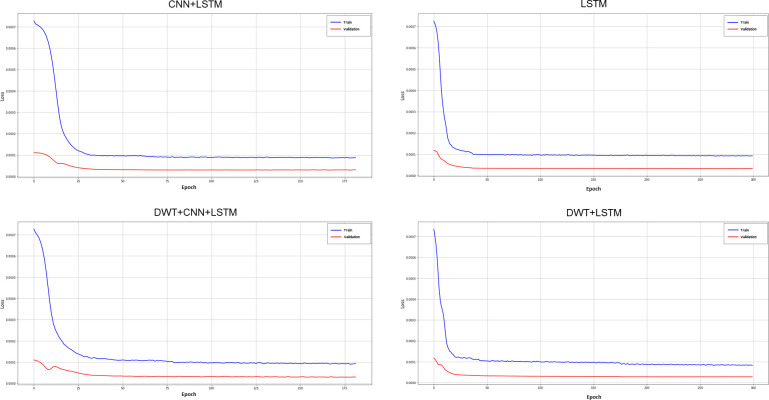
Loss curves for both models and datasets.

Weight optimization was performed using Adam and AdaMax algorithms, for LSTM and CNN+LSTM models, respectively. The Mean Squared Error (MSE) was used as loss function. A feature selection using RandomizedSearchCV, which is a randomized search method for parameter optimization, was applied for hyperparameter tuning. As a result, few hyper-parameters needed adjusting for achieving the best values, as the Adam and AdaMax algorithms usually require little tuning [33]. For the Adam algorithm, a learning rate of 1e-4 was used, the other hyper-parameters were used with default values (learning_rate = 1e-4, beta_1 = 0.9, beta_2 = 0.999, epsilon = 1e-07). For the AdaMax optimizer, all hyper-parameters were used with default values (learning_rate = 0.001, beta_1 = 0.9, beta_2 = 0.999, epsilon = 1e-07) (see Table 6). The Loss curves during training and validation process for both architectures and datasets (with and without Wavelets signals) are presented in Fig 7.

**Table 6:** Optimized hyper-parameters for each method.

Method	Optmizer	Learning_rate	beta_1	beta_2	Epsilon
LSTM	Adam	1e-4	0.9	0.999	1e-07
CNN+LSTM	AdaMax	0.001	0.9	0.999	1e-07

**Fig 8:**
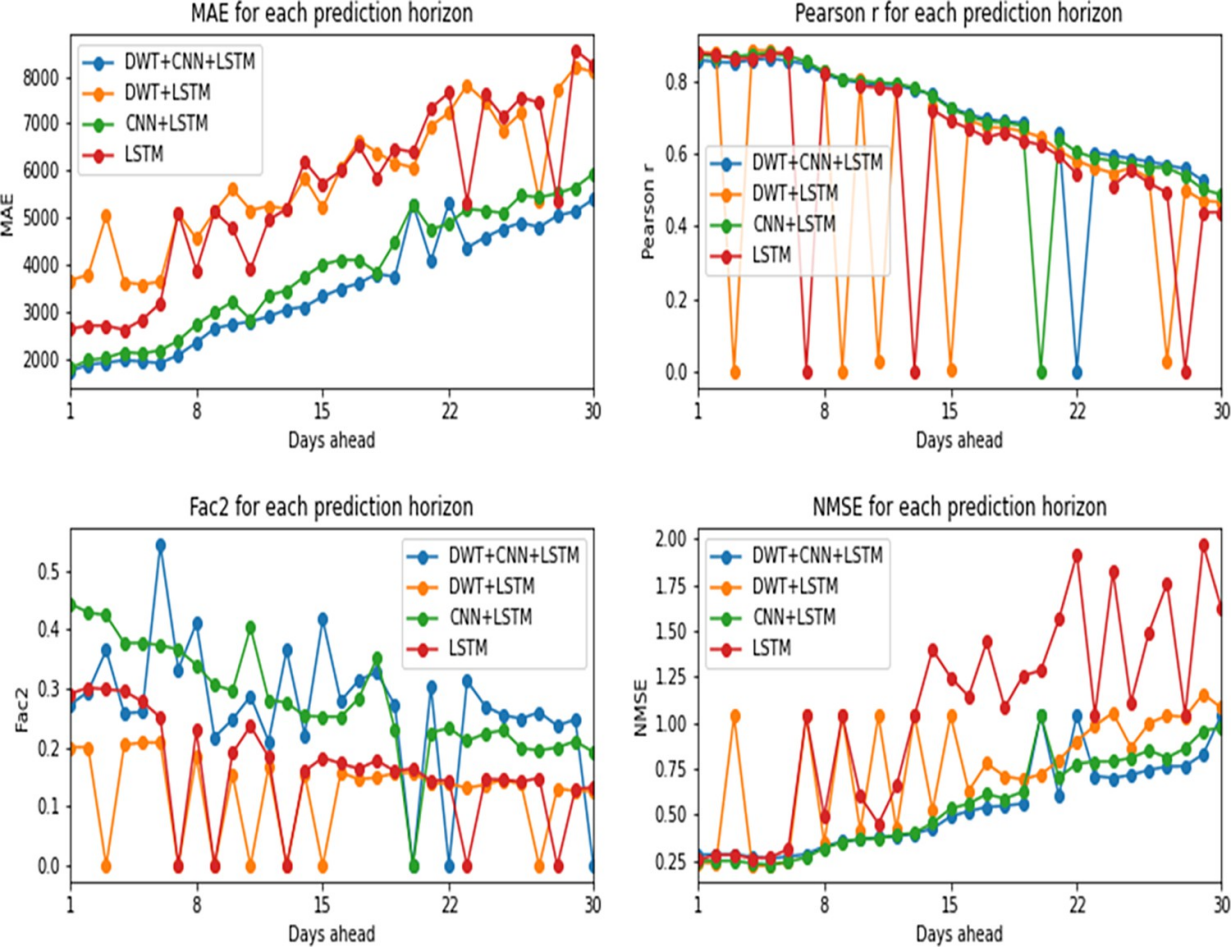
Performance metrics by prediction horizon for confirmed cases.

**Fig 9:**
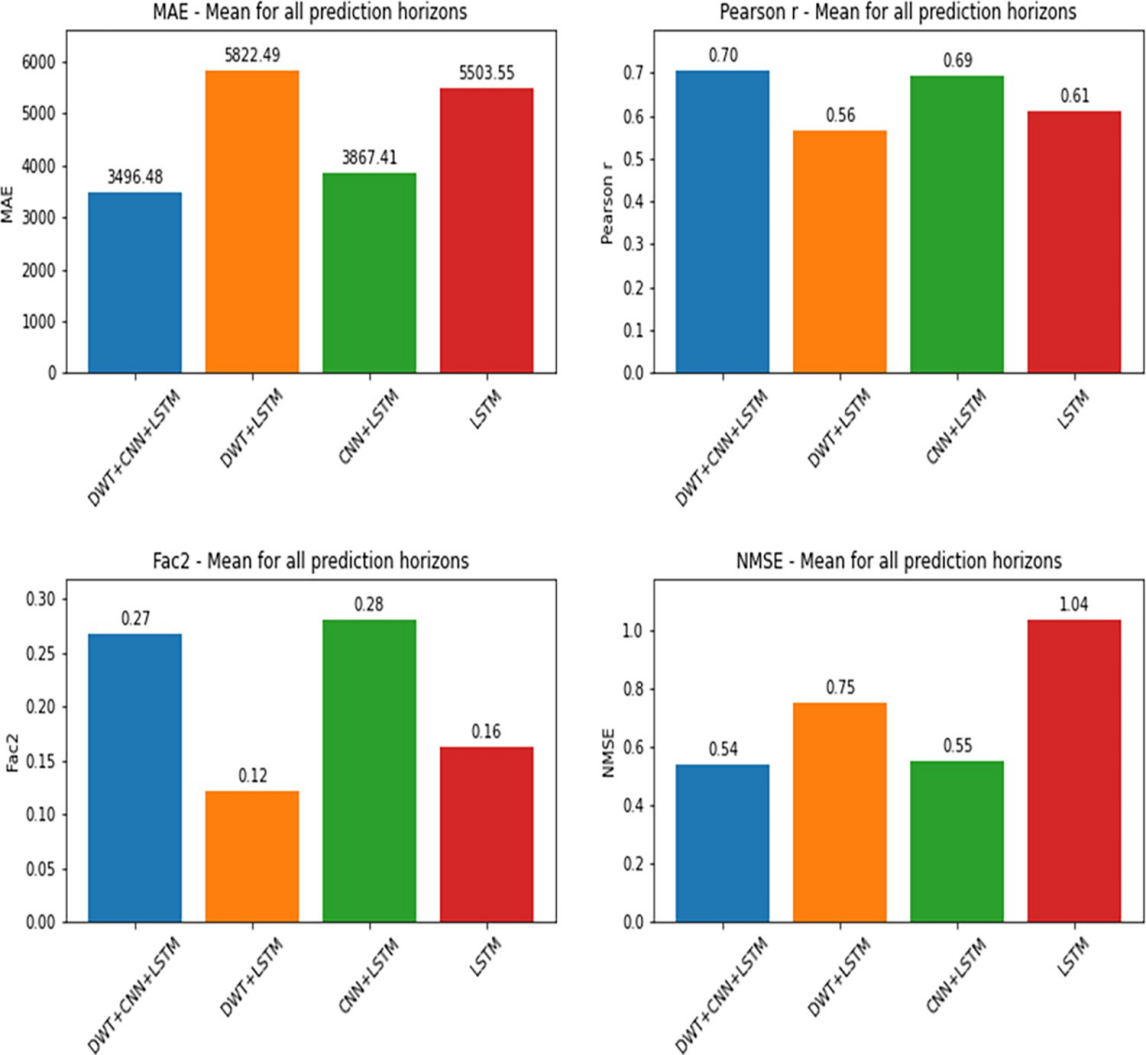
Average metrics from all prediction horizons for confirmed cases, for each deep learning approach.

As can be seen in Figs 8 and 9, confirmed cases prediction was performed with more precision by the hybrid model composed of convolutional and LSTM layers. Considering NMSE, MAE and Pearson correlation coefficient, the version with Wavelets signals (DWT+CNN+LSTM) showed the best results. The Factor of 2 (Fac2) metric indicates that the hybrid model without the addition of Wavelets is the best of all. When comparing LSTM vs. CNN+LSTM in their versions with and without Wavelets separately, it is also noticed the superiority of the hybrid network’s predictive capacity in relation to the LSTM network. That happens because the convolutional layers, combined with the recurrent layers of the LSTM, allow the relationships of the time series to be expressed as different matrices (equivalent to an image) which enables the analysis of a greater amount of information, increasing the learning potential of the network, as explained by Vidal and Kristjanpoller [34].

Commonly applied to computer vision problems, Vidal and Kristjanpoller [34] point out that the combination of convolutional layers with LSTM brings great advantages for the study of time series and is still a little explored approach in this field. The authors argue that the hybrid approach allows a more robust parameter detection, as the network can detect patterns in different perspectives, offering an advantage over most traditional models, which extract information only from the series lags.

Another highlight in the model’s performance is the discrete Wavelets transform application. As it is noticed, there is a performance gain in the models in which Wavelets signals are used as input variables, mainly pointed out by the metrics of error NMSE and MAE, and Pearson R. It is important to remember that this model was trained with only 4 months of data, while the model without wavelet signals was trained with the entire 2020 dataset.

The same is observed in previous studies using coefficients from different families of Wavelets to decompose time series used in predictive modeling of COVID-19 spread [8]. The approach has also been applied to different time series problems with promising results, such as wind speed prediction [24, 25, 35] and solar irradiation prediction [36].

Fig 8 presents the models’ performance metrics for each prediction horizon and Fig 9 presents the metrics mean from prediction horizons. Both are concerned confirmed cases models.

To determine whether there is a statistically significant difference between the models’ performances, we conducted a Kruskal-Wallis Test. A Shapiro-Wilk test was also performed for checking normality, which indicated that the data were not normally distributed considering a 0.05 significance level (p-value = 1.171e-06). We compared the medians of NMSE from each approach for testing the null hypothesis that the median is equal across all models. The test statistic was 22.26 and the corresponding p-value was 5.759e-05, which means that we reject the null hypothesis that the median is the same. With sufficient evidence to conclude that the different deep learning approaches leads to statistically significant differences in prediction, we then conducted Nemenyi post hoc test to calculate pairwise comparisons. Nemenyi results can be observed in Table 7, which points to significant differences between the LSTM and CNN+LSTM models, and between LSTM and DWT+CNN+LSTM.

**Table 7:** Pairwise comparisons from Nemenyi’s all-pairs test with chi-square approximation for confirmed cases models.

	CNN+LSTM	DWT+CNN+LSTM	DWT+LSTM
DWT+CNN+LSTM	0.99996	-	-
DWT+LSTM	0.25237	0.27539	-
LSTM	0.00093	0.00113	0.24792

**Fig 10:**
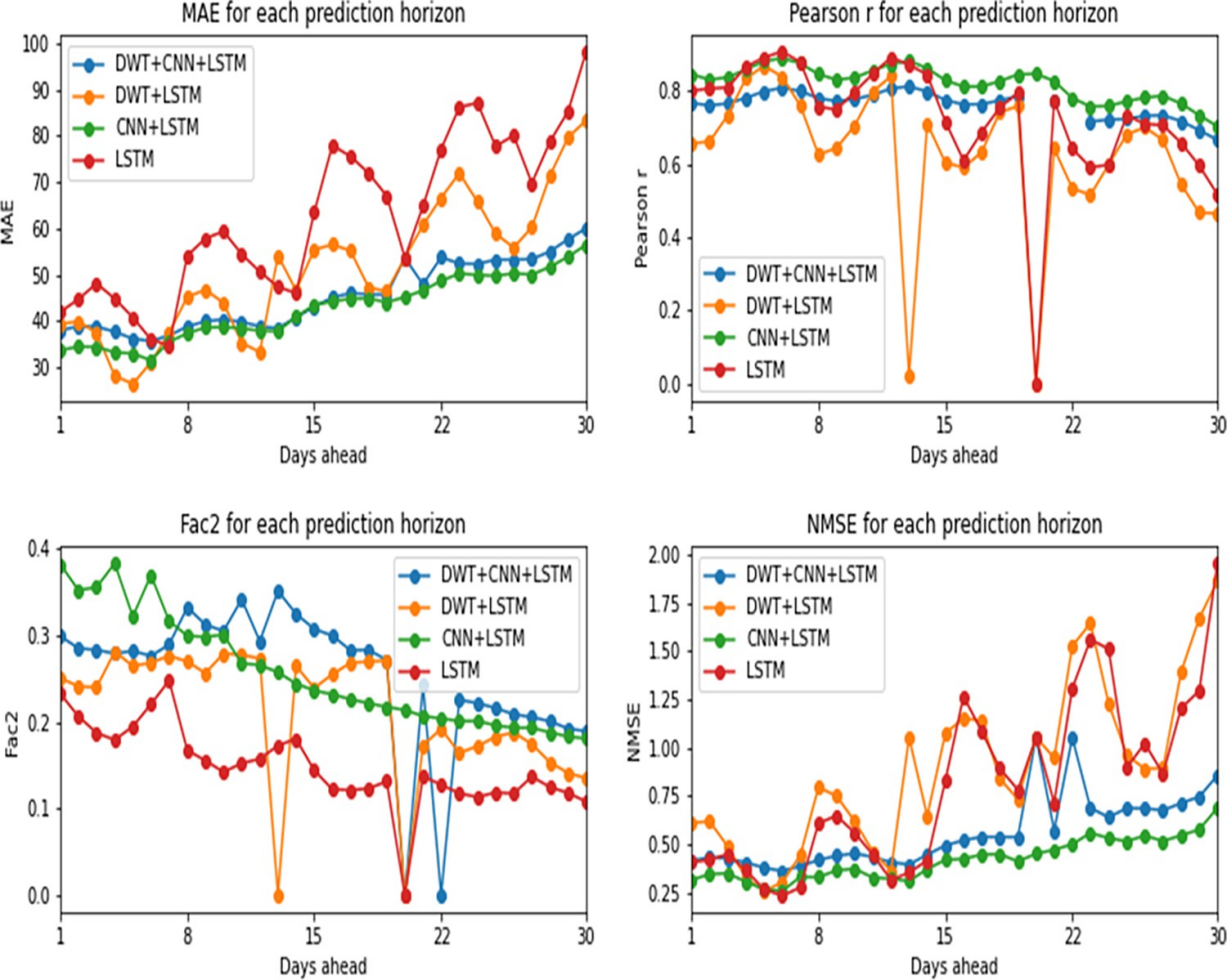
Performance metrics by prediction horizon for deaths.

**Fig 11:**
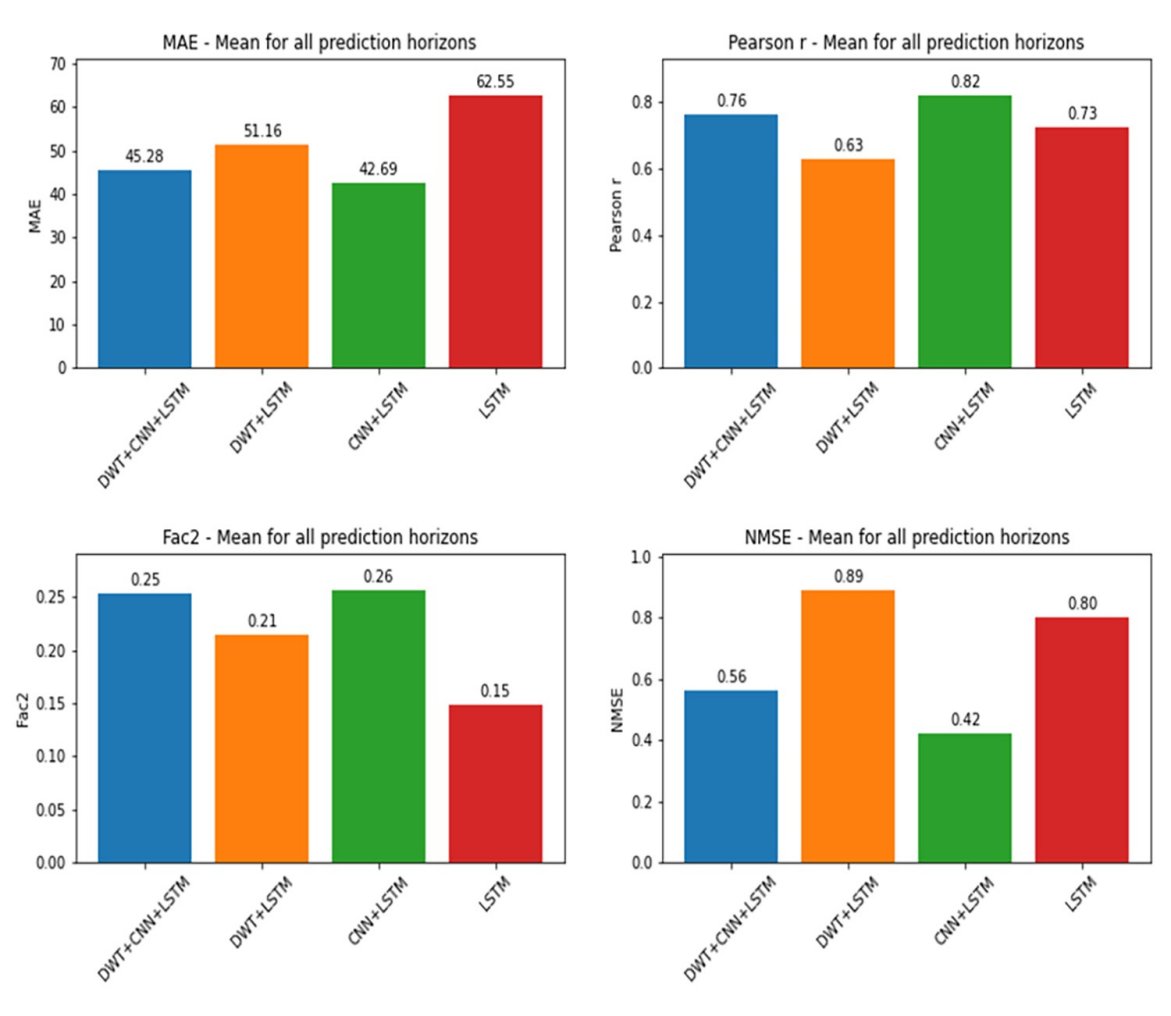
Average metrics from all prediction horizons for deaths, for each deep learning approach.

For the prediction of daily death values, on the other hand, CNN+LSTM hybrid model without Wavelets presented the best performances, according to the metrics. All the results can be seen in Figs 10 and 11.

The Kruskal-Wallis Test for deaths prediction models indicated statistically significant differences between performances from a NMSE analysis, with a test statistic of 30.232 and corresponding p-value of 1.233e-06. A Shapiro-Wilk normality test presented a p-value of 1.747e-09, indicating that the data were not normally distributed. Pairwise comparisons from Nemenyi post hoc test points significant differences between the DWT+LSTM and CNN+LSTM, and DWT+LSTM and DWT+CNN+LSTM, and between LSTM and CNN+LSTM (Table 8).

**Table 8:** Pairwise comparisons from Nemenyi’s all-pairs test with chi-square approximation for deaths.

	CNN+LSTM	DWT+CNN+LSTM	DWT+LSTM
DWT+CNN+LSTM	0.1296	-	-
DWT+LSTM	6.4e-06	0.0492	-
LSTM	0.0011	0.4462	0.7130

Despite the divergences between these results, it is possible to observe that in all cases the hybrid architecture CNN+LSTM presented better performance than the LSTM model. It is important to remember that despite being trained separately, both modellings (confirmed cases and deaths) were performed with the same architectures, data sets and input variables, the target being the only difference between them. Therefore, the explanation for this divergence between the results possibly lies in each output variable time series.

In order to carry out a qualitative evaluation of the hybrid model DWT+CNN+LSTM performance, we used the trained model to infer values of 30 time-steps for countries in each of the five inhabited continents, considering North and South America separately. Six countries were selected and analyzed, namely: India (Asia), South Africa (Africa), United States (North America), Brazil (South America), United Kingdom (Europe), and Australia (Oceania). After the inference, we used NMSE values to select the best prediction horizon for each country. We then analyzed the other metrics, as well as the time series built using the model and the dispersion/association of the predicted data compared to the real data. The analyzes were performed with data from January/2021 to March/2022. The process was carried out for the confirmed cases model and for the deaths model using the same countries as a basis.

For inferences performed by the confirmed cases model, four prediction horizons showed the best metrics, considering NMSE (lowest values): t+1 (United Kingdom, Australia, South Africa), t+2 (India), t+5 (United States), t+6 (Brazil). The best NMSE from a prediction horizon among the countries under analysis was presented by India, with 0.1185 for the t+2 horizon. India also presented the best Pearson R. The best Factor of 2 were presented by the United Kingdom, for the t+1 horizon. And the best absolute error was presented by Australia, for the t+1 horizon. All values can be verified in Table 9.

**Table 9:** Metrics—top prediction horizon for confirmed cases by country.

Country	Prediction horizon	NMSE	Pearson R	Factor of 2	MAE
	(best NMSE)				
United Kingdom	t+1	0.5505	0.6712	0.9716	8718.97
India	t+2	0.1185	0.9652	0.9576	19911
Australia	t+1	0.1310	0.9390	0.3714	219.93
South Africa	t+1	0.2911	0.8561	0.7345	2321.71
Brazil	t+6	0.3194	0.8363	0.8630	16078.1
United States	t+5	0.5365	0.7707	0.8390	62439.9

**Fig 12:**
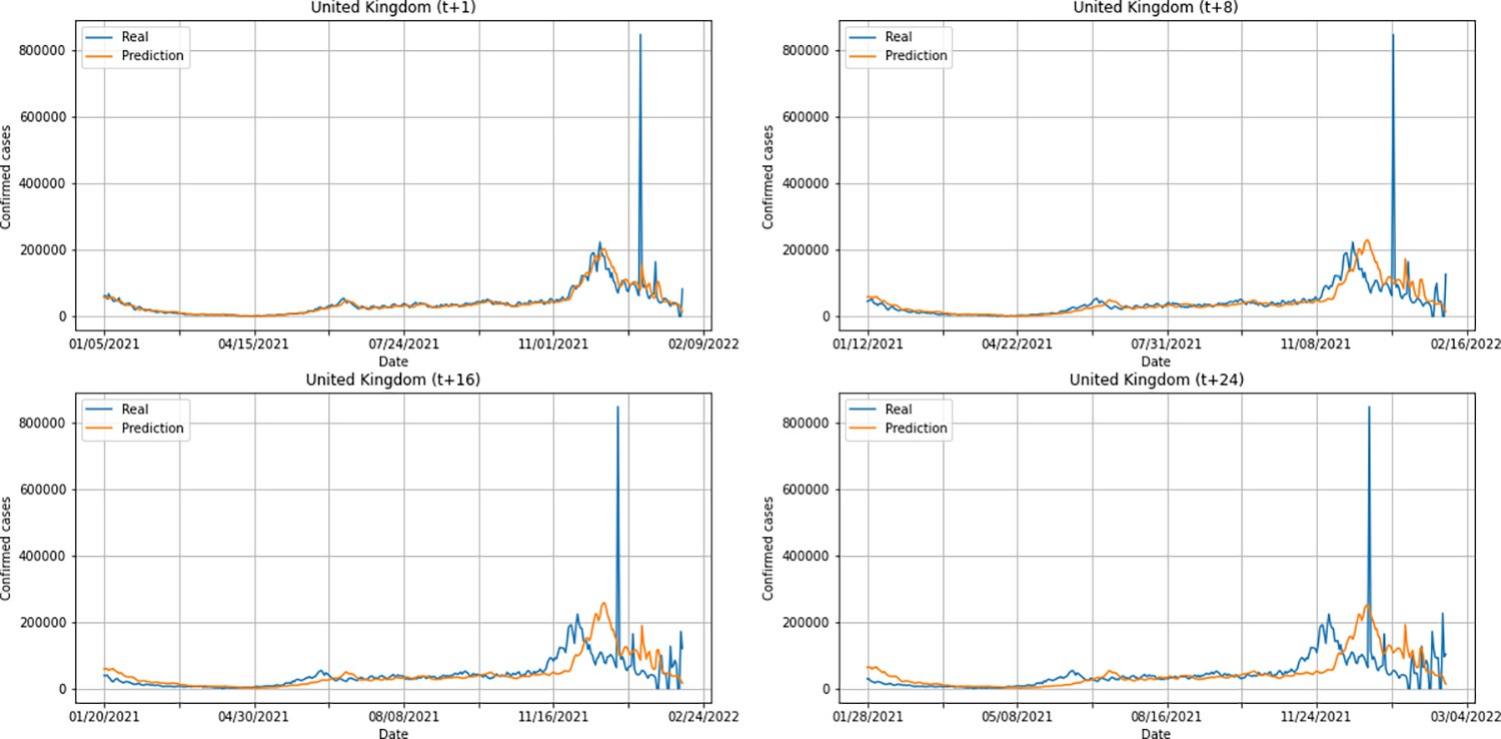
Predictions for confirmed cases–United Kingdom.

**Fig 13:**
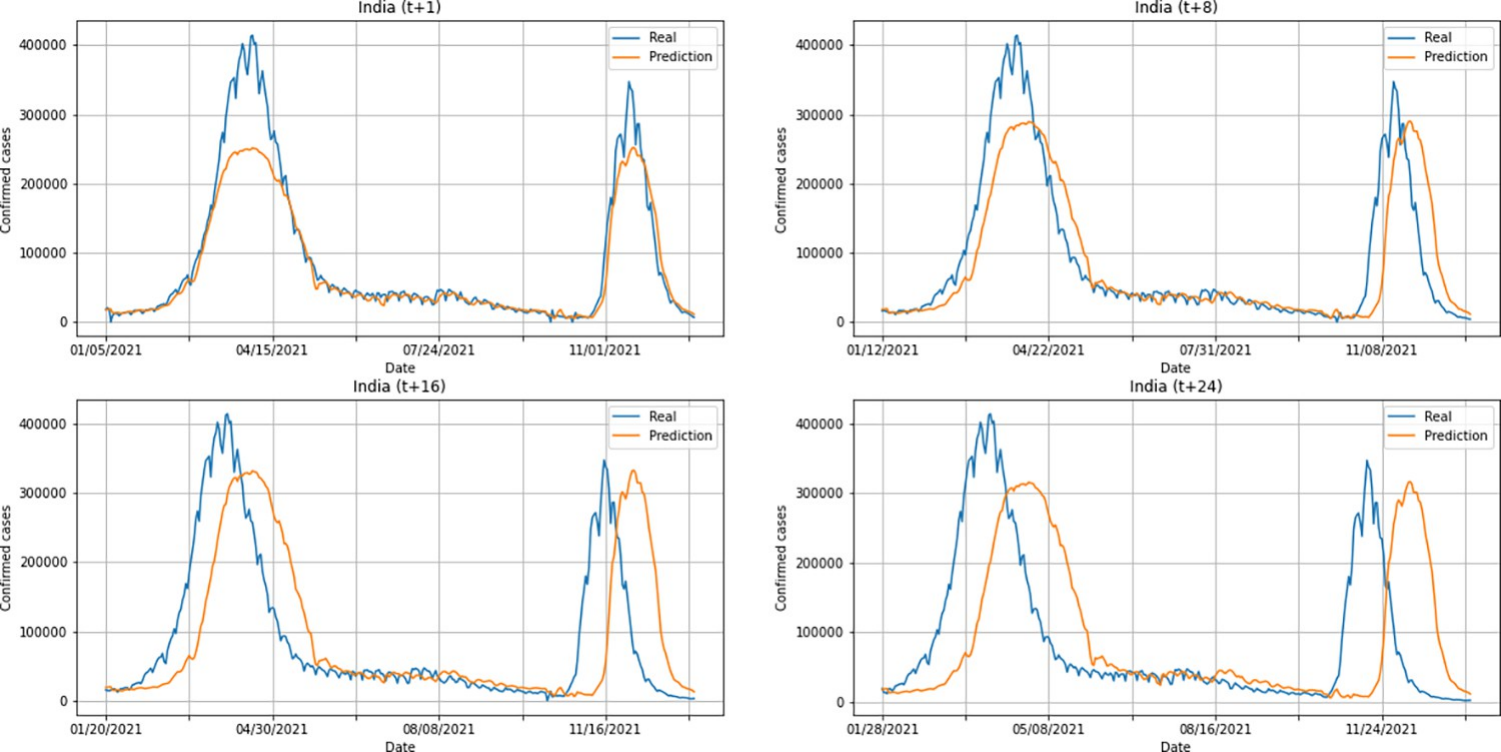
Predictions for confirmed cases–India.

**Fig 14:**
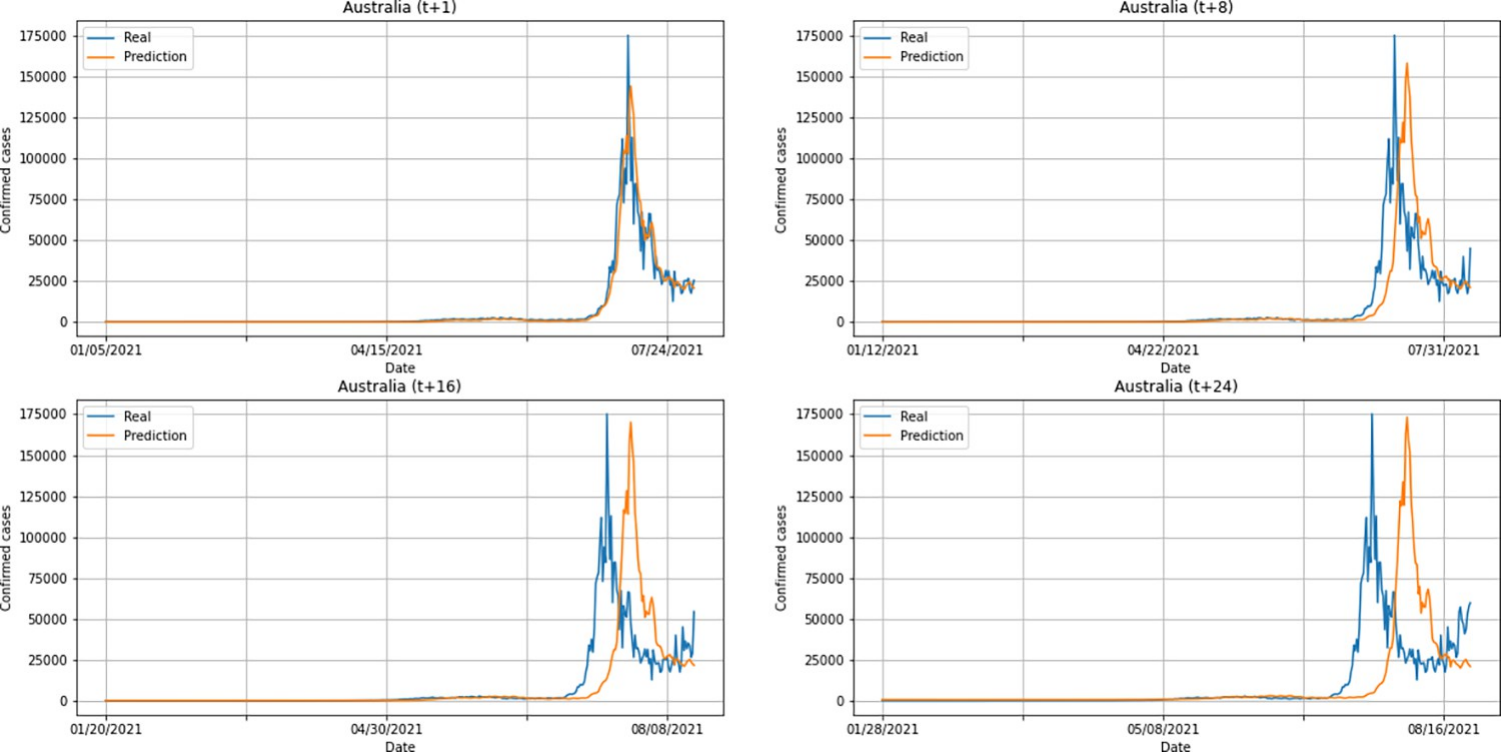
Predictions for confirmed cases–Australia.

**Fig 15:**
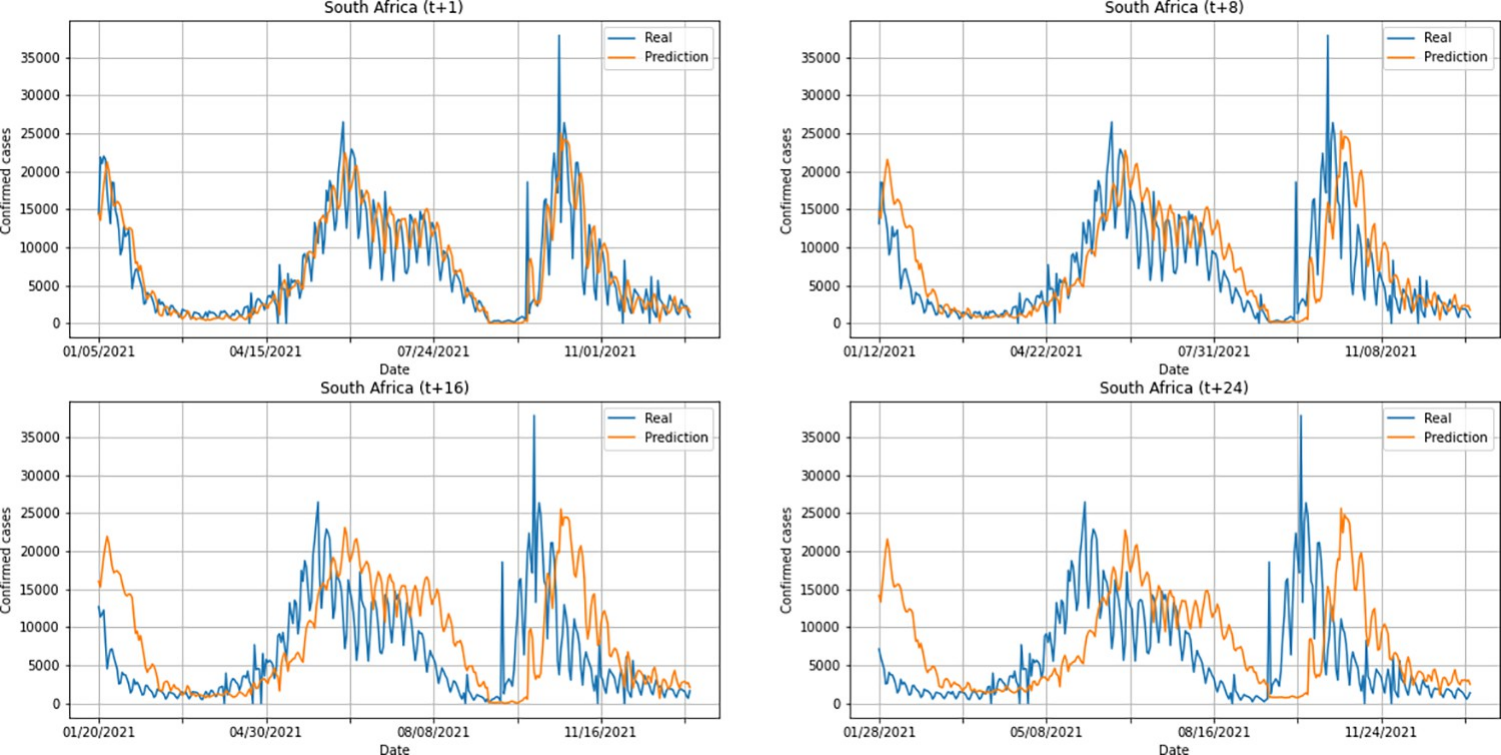
Predictions for confirmed cases–South Africa.

**Fig 16:**
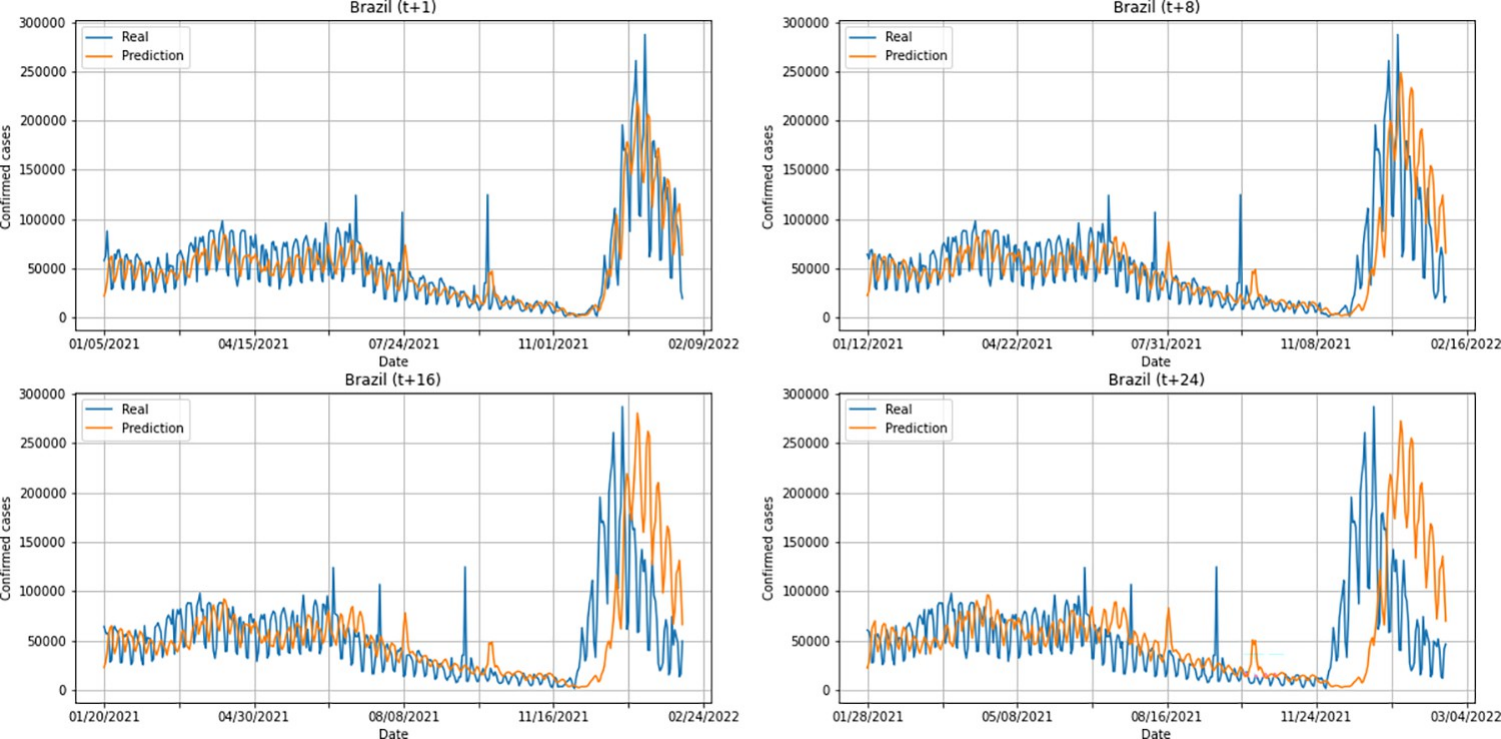
Predictions for confirmed cases–Brazil.

**Fig 17:**
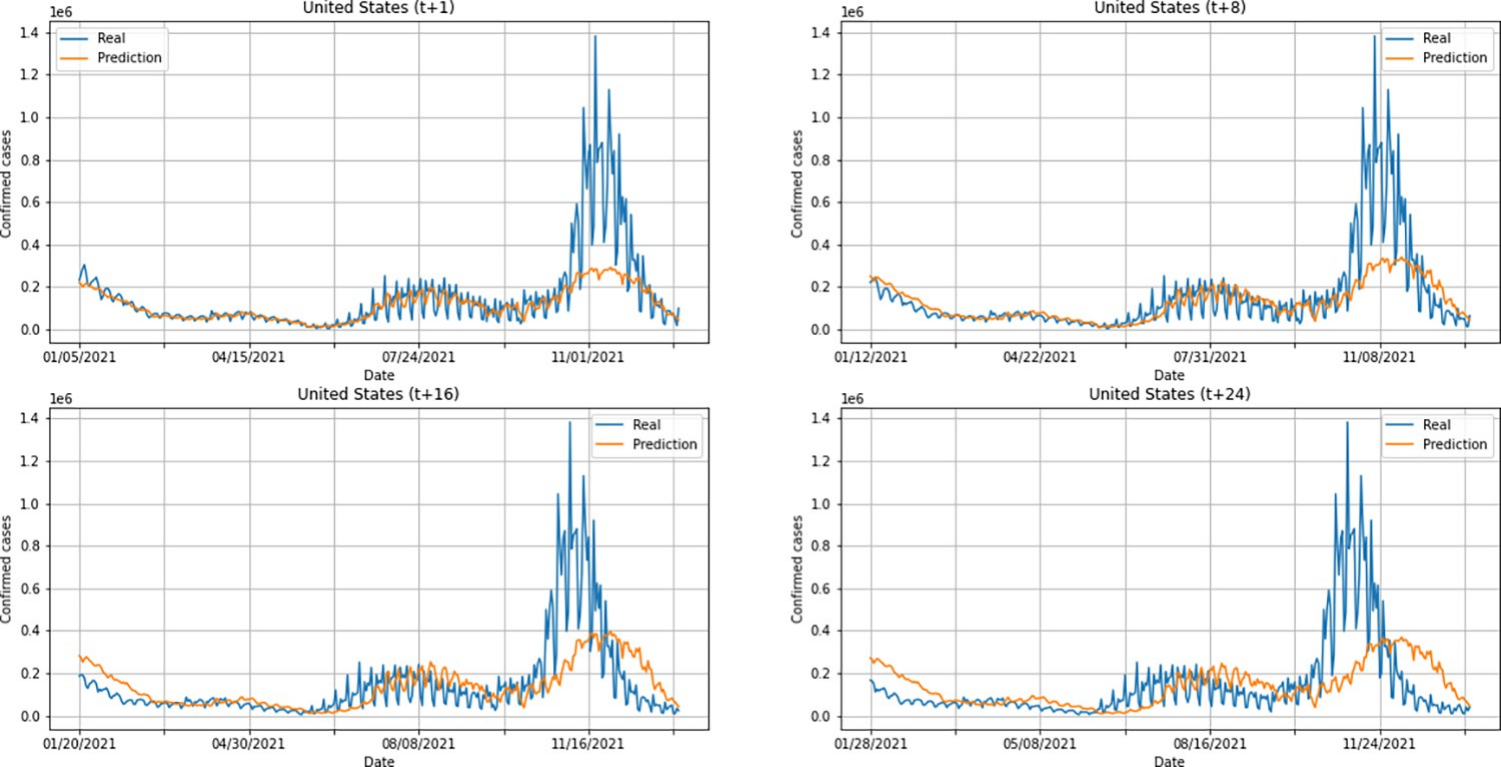
Predictions for confirmed cases–United States.

A graphical analysis of the time-series for confirmed cases constructed by the model, considering the evolution of the prediction horizons in each country shows that, in general, the model succeeds to follow trends in the original time-series behavior, with some apparent shifts. In all cases, the t+1 series presented the best similarity to the real data. The graphs presenting an evolution from four time-steps (t+1, t+8, t+16, t+24) for each of the six countries under analysis can be seen in Figs 12–17.

**Fig 18:**
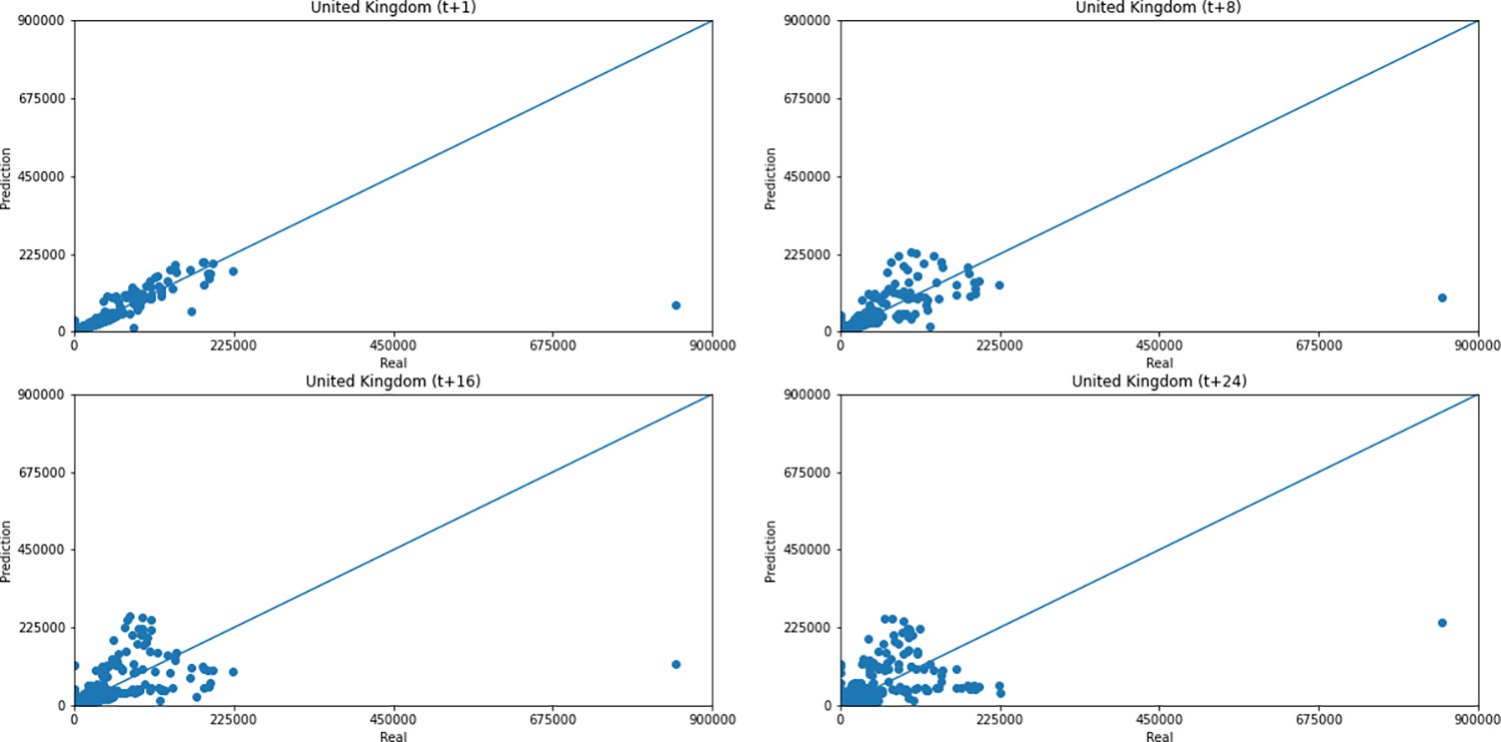
Scatter plot—predicted values vs. actual values for confirmed cases–United Kingdom.

**Fig 19:**
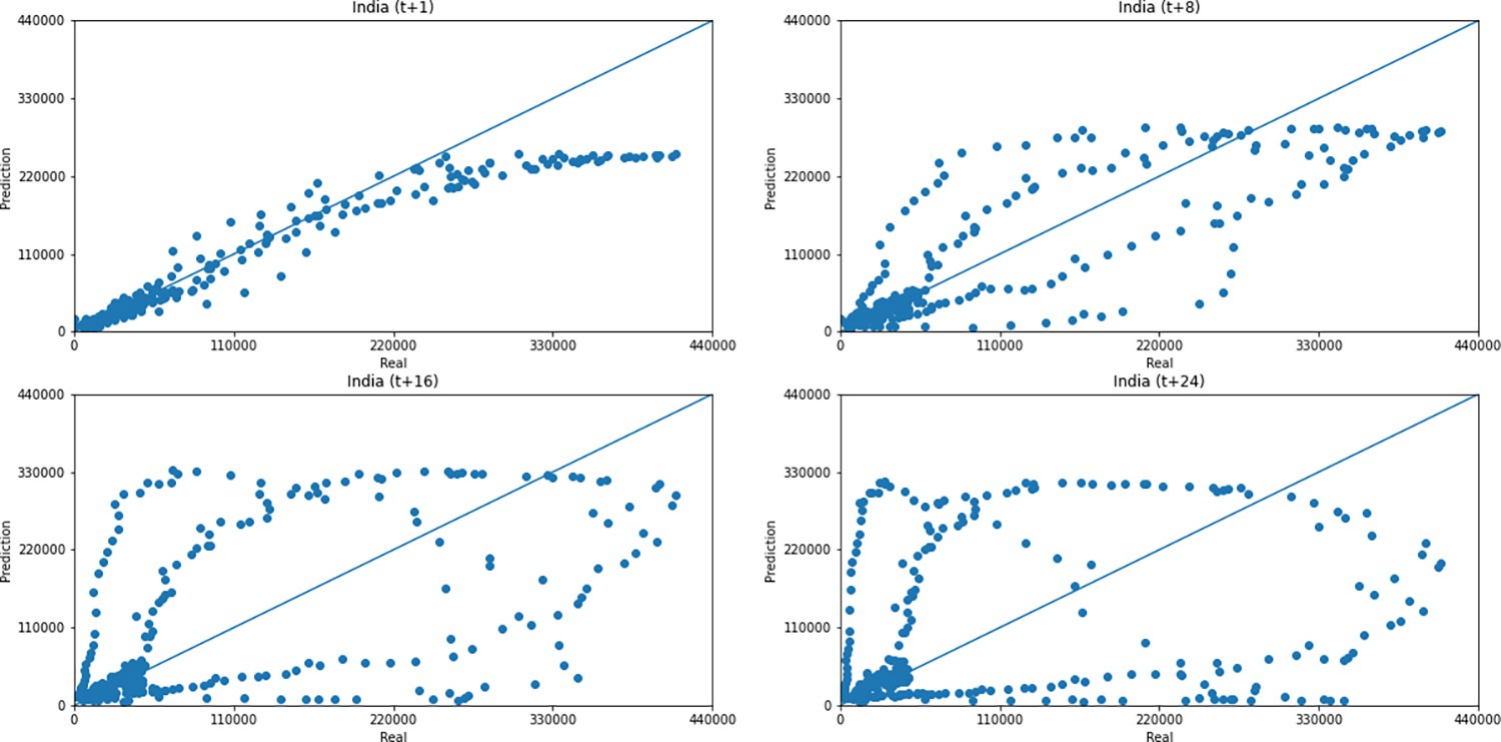
Scatter plot—predicted values vs. actual values for confirmed cases–India.

**Fig 20:**
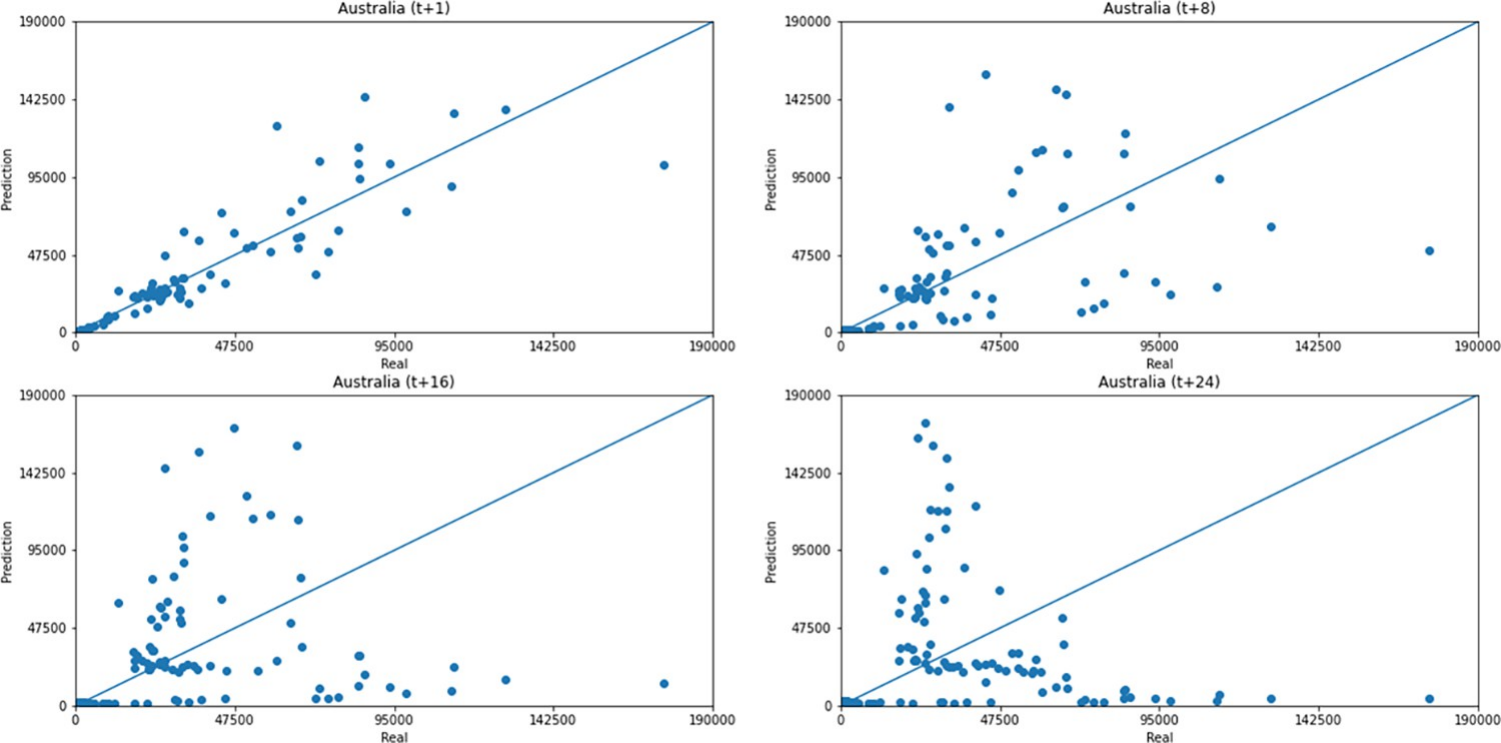
Scatter plot—predicted values vs. actual values for confirmed cases–Australia.

**Fig 21:**
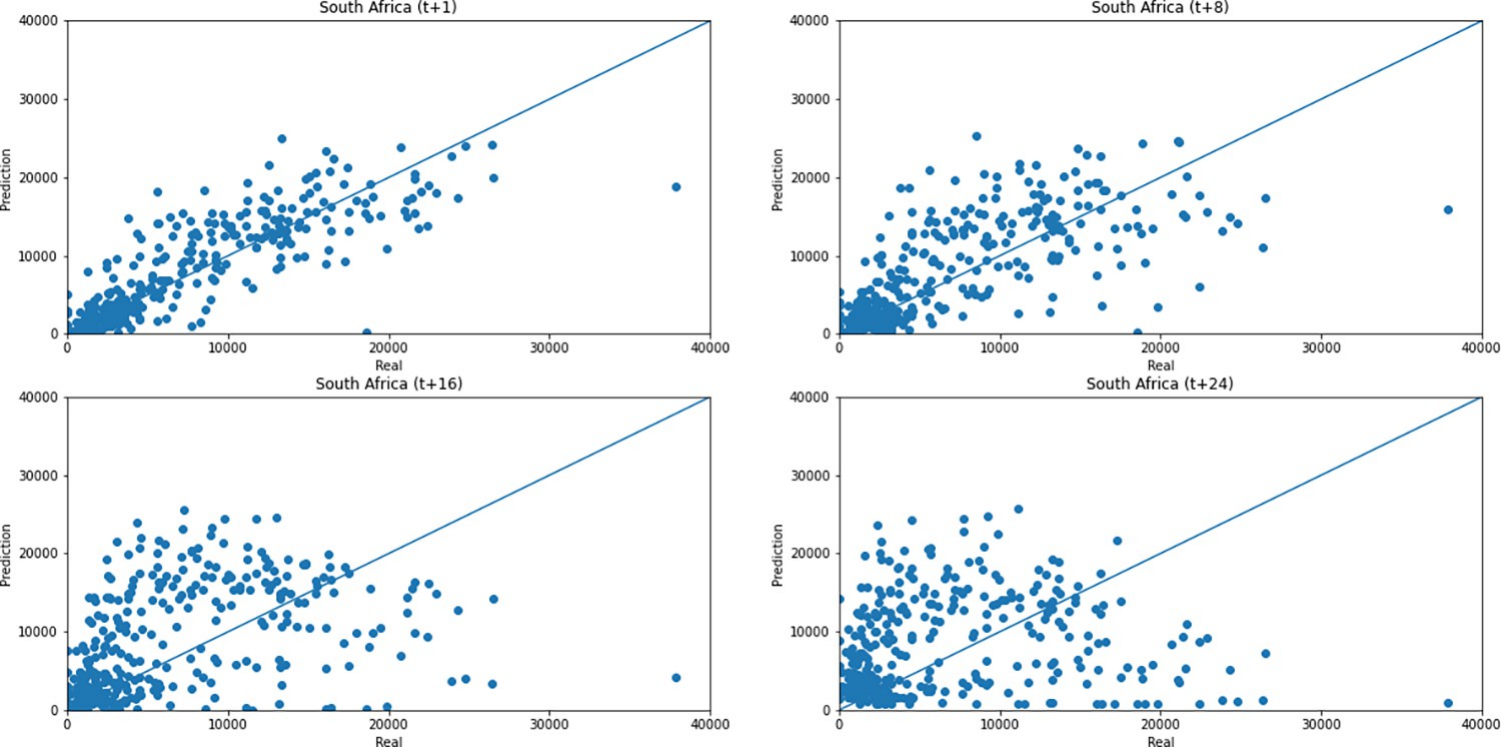
Scatter plot—predicted values vs. actual values for confirmed cases–South Africa.

**Fig 22:**
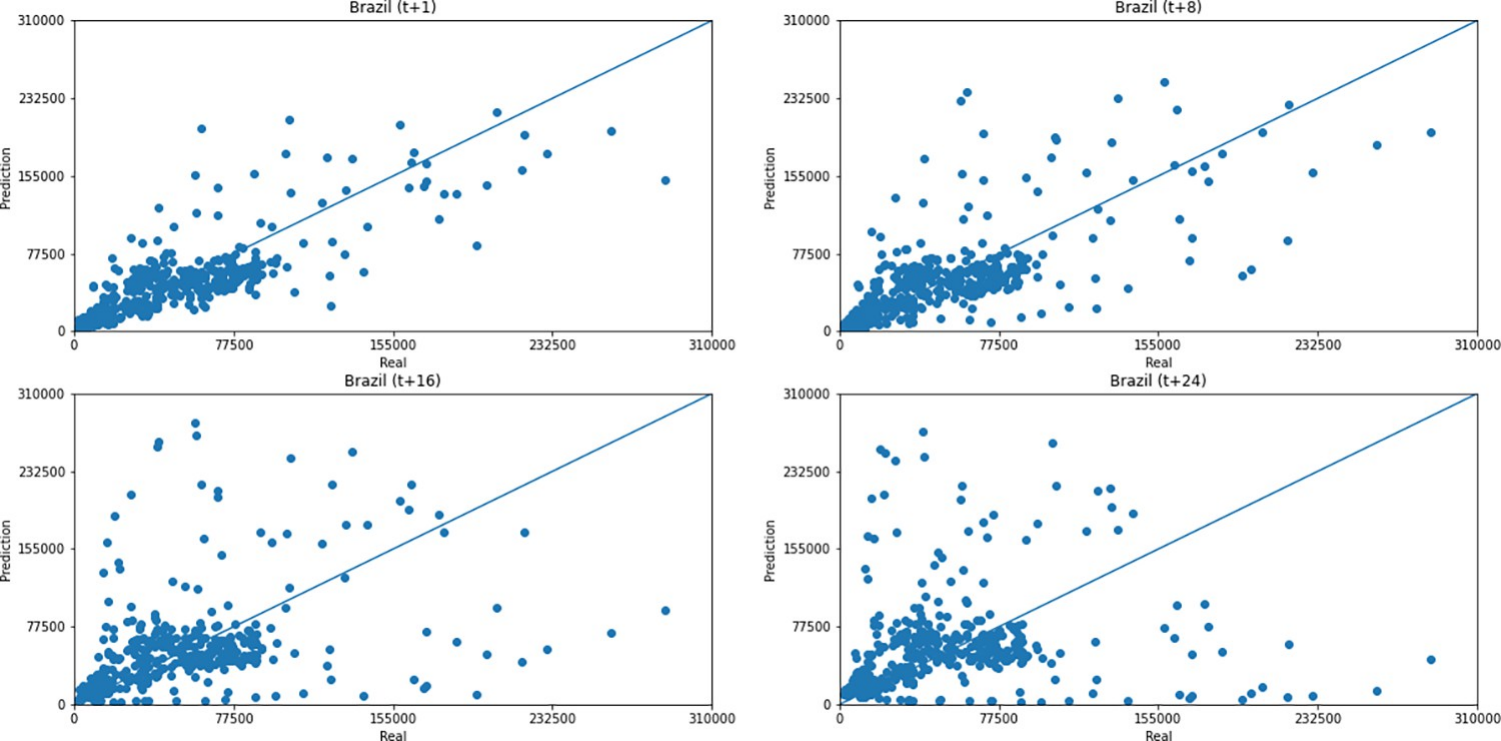
Scatter plot—predicted values vs. actual values for confirmed cases–Brazil.

**Fig 23:**
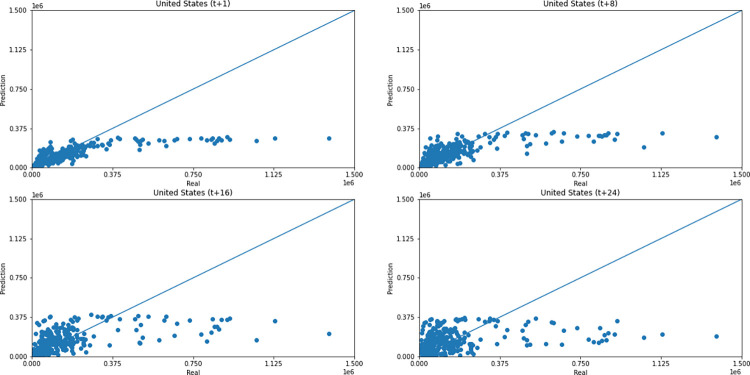
Scatter plot—predicted values vs. actual values for confirmed cases–United States.

As for a data dispersion analysis, we use scatter plots to observe relationships between predicted data and real. All graphics can be seen in Figs 18–23. The graph that seems to best approximate linear behavior is the scatterplot between the actual and predicted data for Australia and for South Africa, both for prediction horizon t+1. The graph analysis corroborates with the metrics, since the Pearson R values, which measures the association between the data, for Australia and for South Africa were 0.9390 and 0.8561, respectively. Although India has a higher Pearson R, there is no linear behavior of association between the data, which would require further studies to explain the dependence between them.

Regarding the inferences performed by the model for the prediction of deaths, different prediction horizons showed the best metrics: t+1 (United Kingdom), t+6 (United States, South Africa), t+9 (India), t+13 (Brazil), t+20 (Australia). It is interesting to notice that, different from confirmed cases models, horizons further away from the last real data also appeared here, as is the case with t+13 and t+20. The best NMSE from a prediction horizon among the countries under analysis was presented by India, with 0.2460 for the t+9 horizon. The best Pearson R was also presented by India. Best Factor of 2 was presented by the United States and the best absolute error was presented by South Africa, both for the t+6 horizon. All values can be verified in Table 10.

**Table 10:** Metrics—top prediction horizon for deaths by country.

Country	Prediction horizon	NMSE	Pearson R	Factor of 2	MAE
	(best NMSE)				
India	t+9	0.2460	0.8715	0.7825	336.106
United States	t+6	0.4915	0.7157	0.7966	557.537
South Africa	t+6	0.3810	0.7906	0.7373	75.0377
Brazil	t+13	0.5798	0.7218	0.7881	480.529
United Kingdom	t+1	1.4686	0.3712	0.2877	308.66
Australia	t+20	1.3177	0	0	11.5095

**Fig 24:**
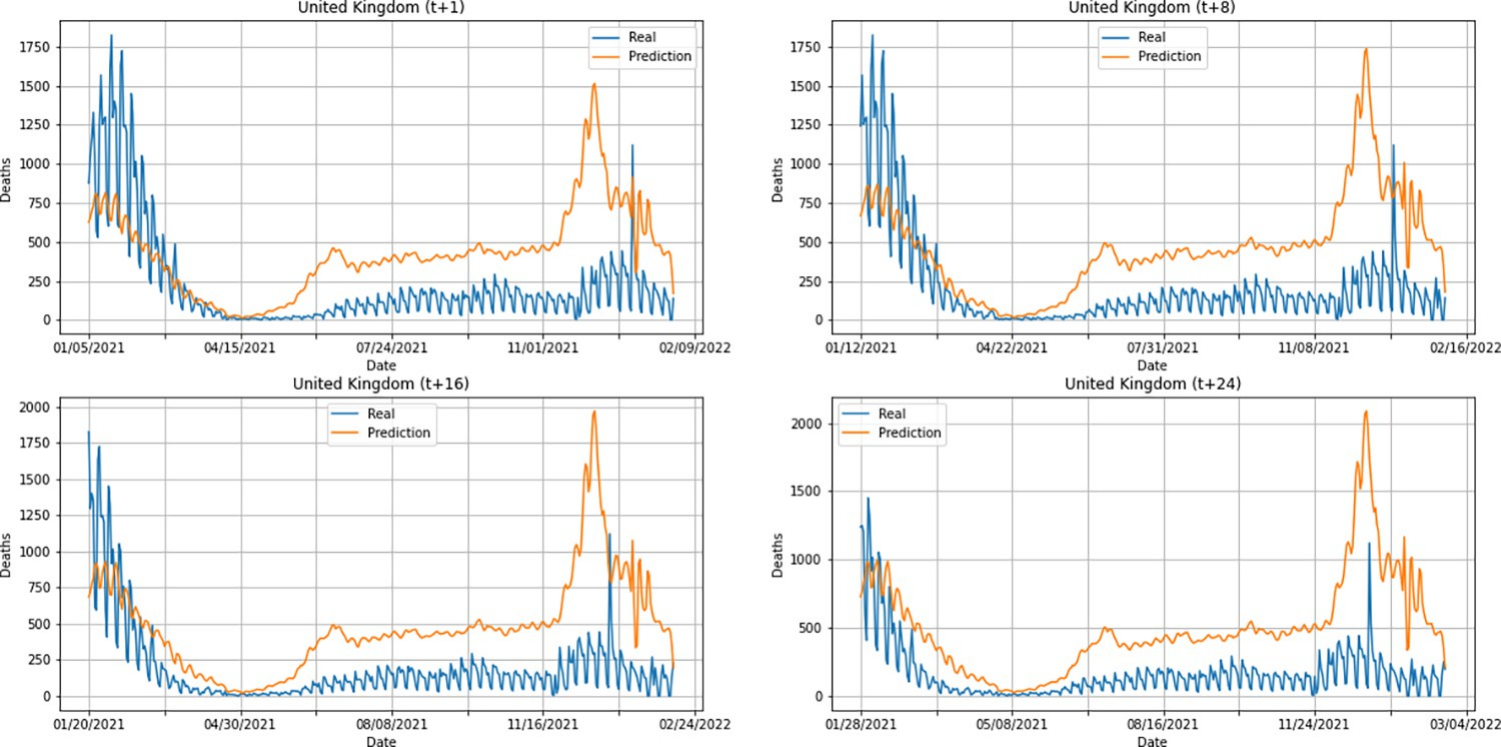
Predictions for confirmed deaths–United Kingdom.

**Fig 25:**
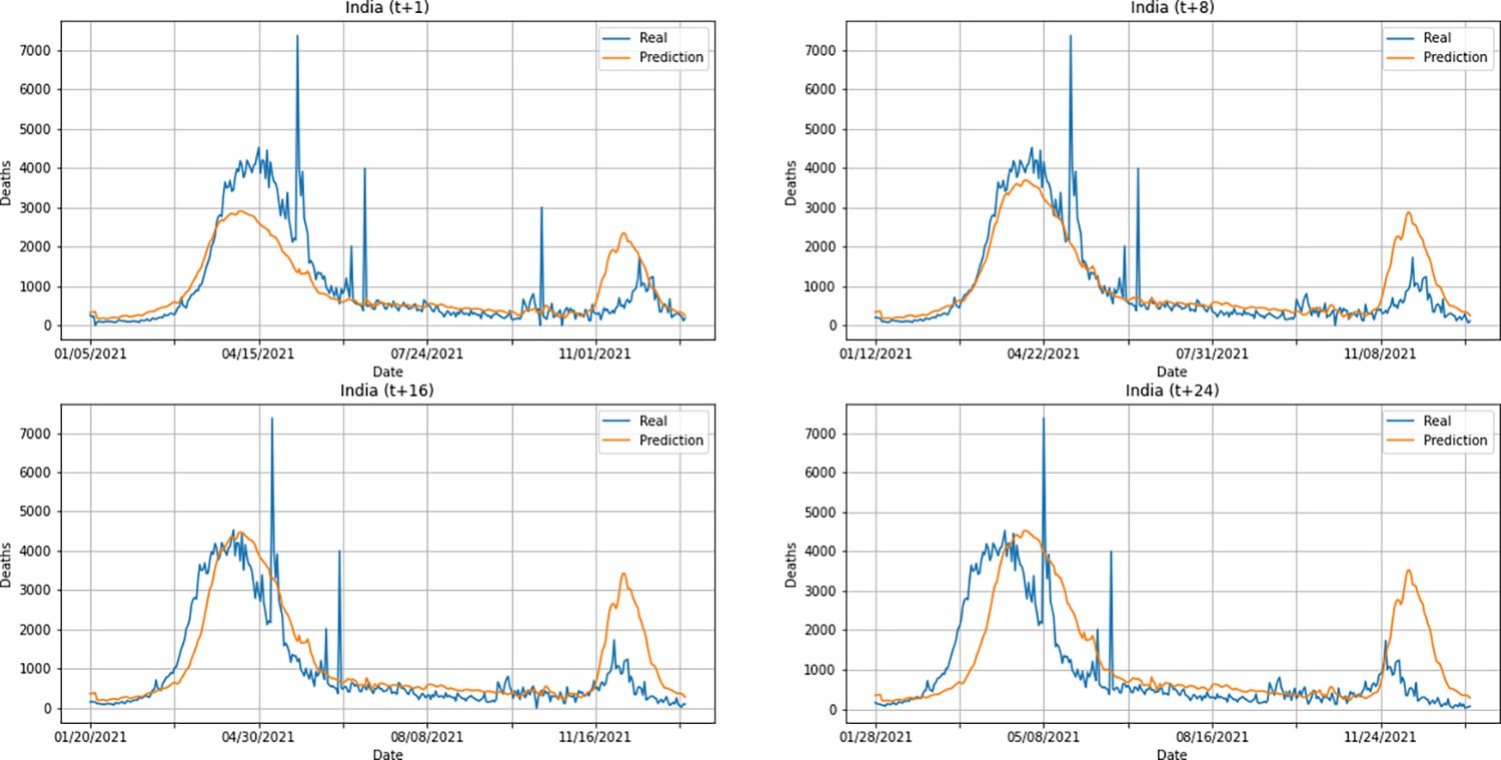
Predictions for confirmed deaths–India.

**Fig 26:**
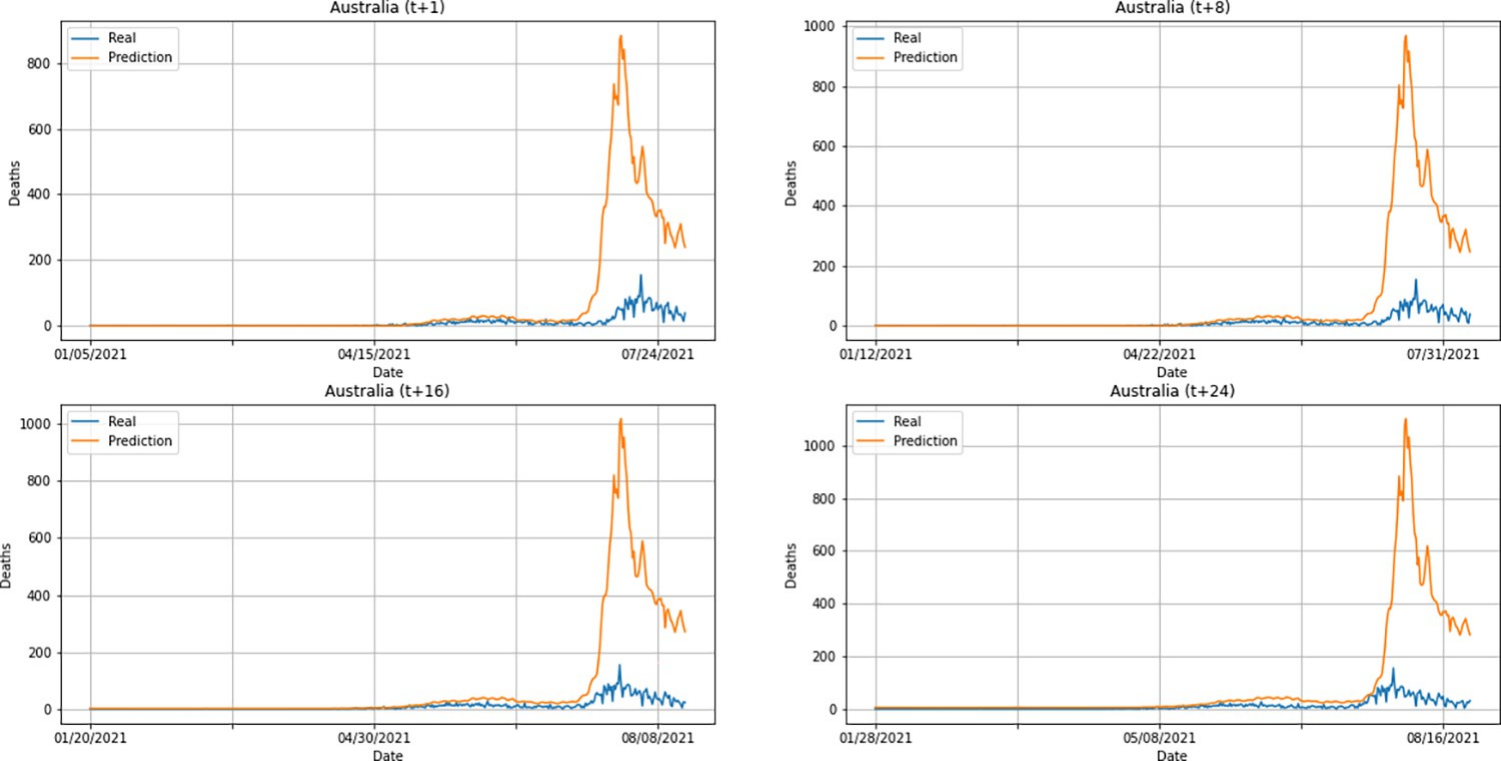
Predictions for confirmed deaths–Australia.

**Fig 27:**
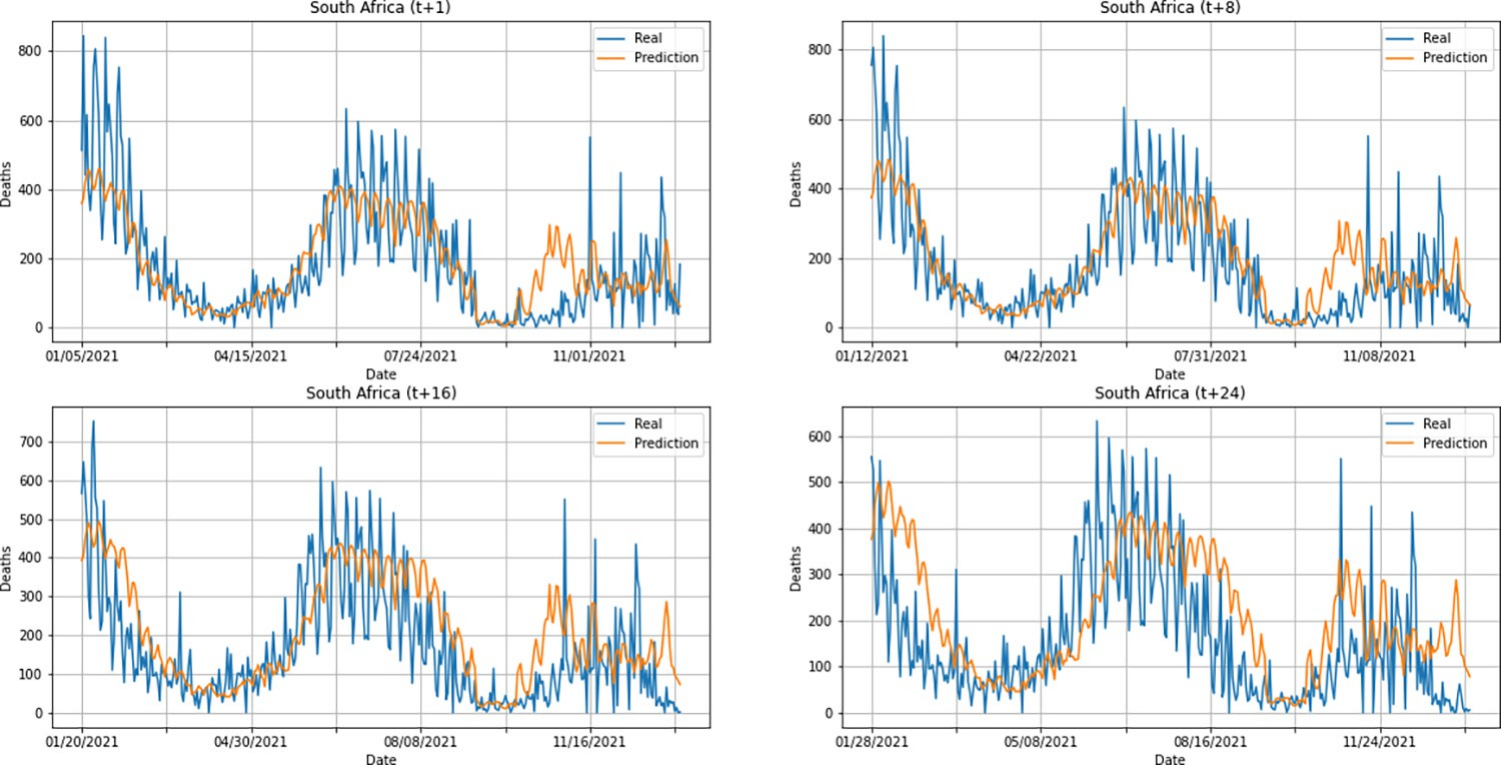
Predictions for confirmed deaths–South Africa.

**Fig 28:**
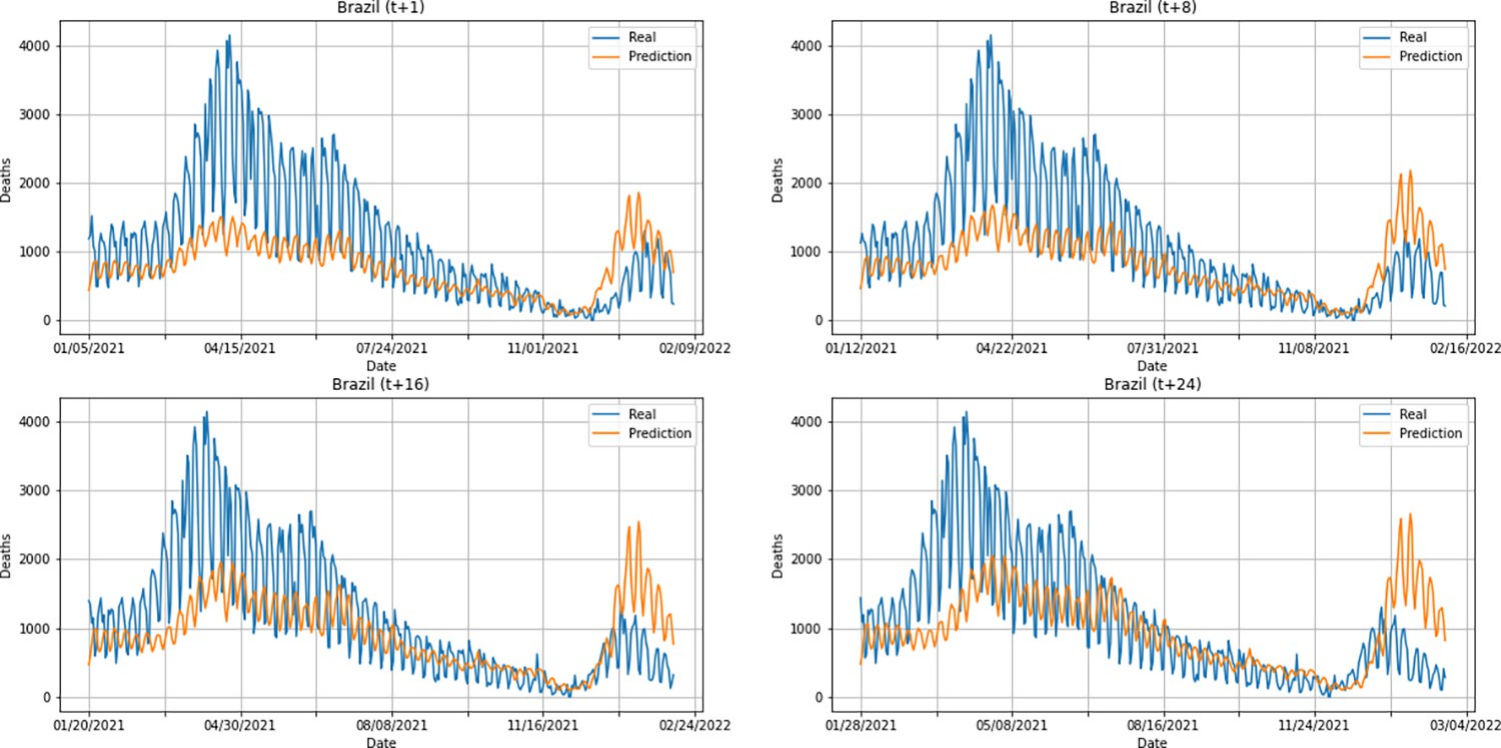
Predictions for confirmed deaths–Brazil.

**Fig 29:**
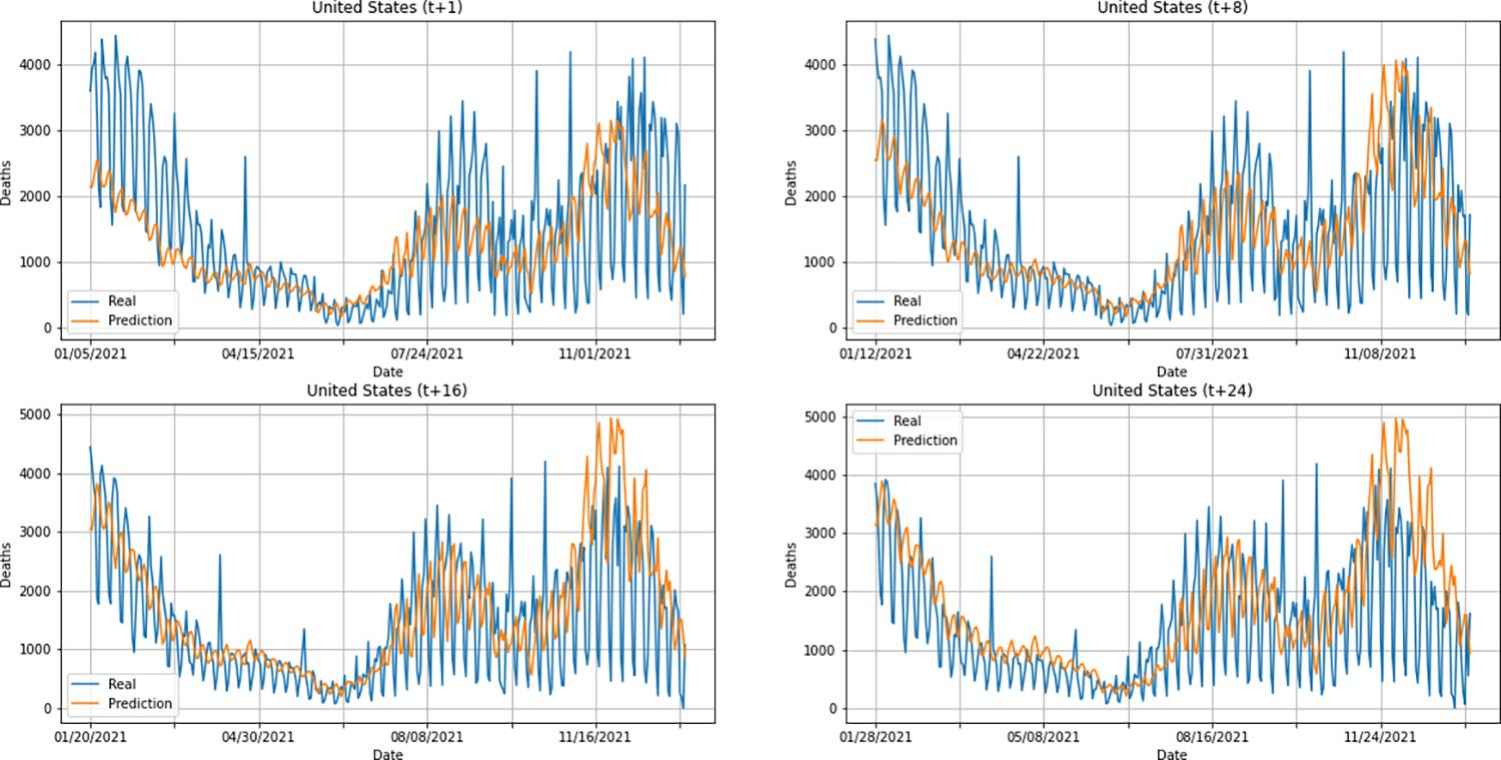
Predictions for confirmed deaths–United States.

From a graphical analysis of the time-series for deaths, it is possible to notice that some series constructed by the model for the prediction horizons selected exceeded the real values. This is the case of the United Kingdom and Australia, as can be seen in Figs 14 and 26. The series built for India presented the best similarity to the real data, considering the prediction horizons evolution. As with the confirmed cases model, this corroborates the fact that India presented the best NMSE value in one of its prediction horizons. The graphs below (Figs 24–29) present the deaths prediction evolution from four time-steps (t+1, t+8, t+16, t+24) for each of the six countries under analysis.

The graphs of confirmed cases and deaths presented in our analysis reveal that the model identified, even with low precision in some cases, a new wave of COVID-19 infection in late 2021 and early 2022 for all countries analyzed. This period corresponds to the registration of the emergence of Omicron, carried out for the first time in November 2021 by South Africa. The variant was quickly identified by governments worldwide and, in a short time, became dominant with a high number of new infections on a daily basis, which was also predicted by our model. Thus, we verified the potential of our model for the prediction of pandemic behavior, including new infection waves from different variants of SARS-CoV-2.

**Fig 30:**
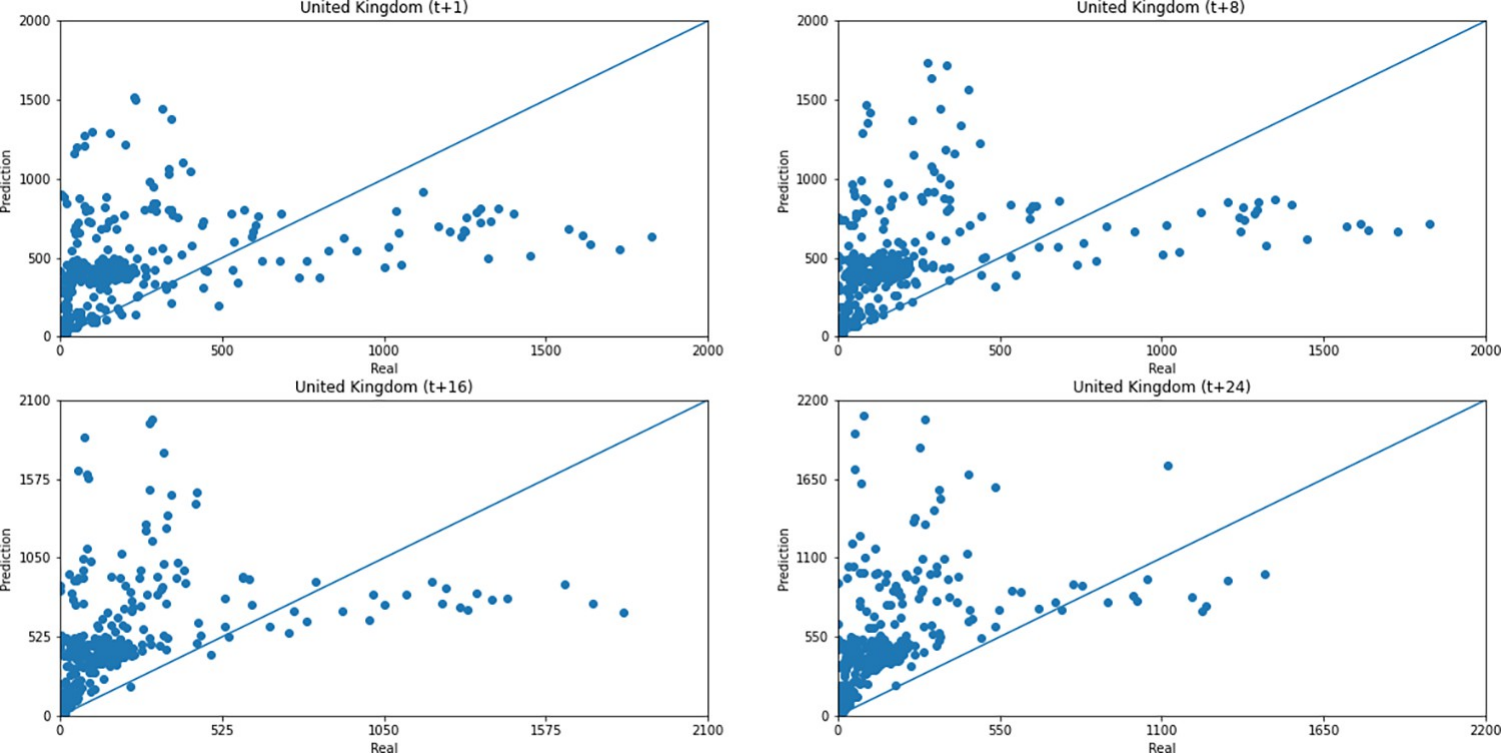
Scatter plot—predicted values vs. actual values for confirmed deaths–United Kingdom.

**Fig 31:**
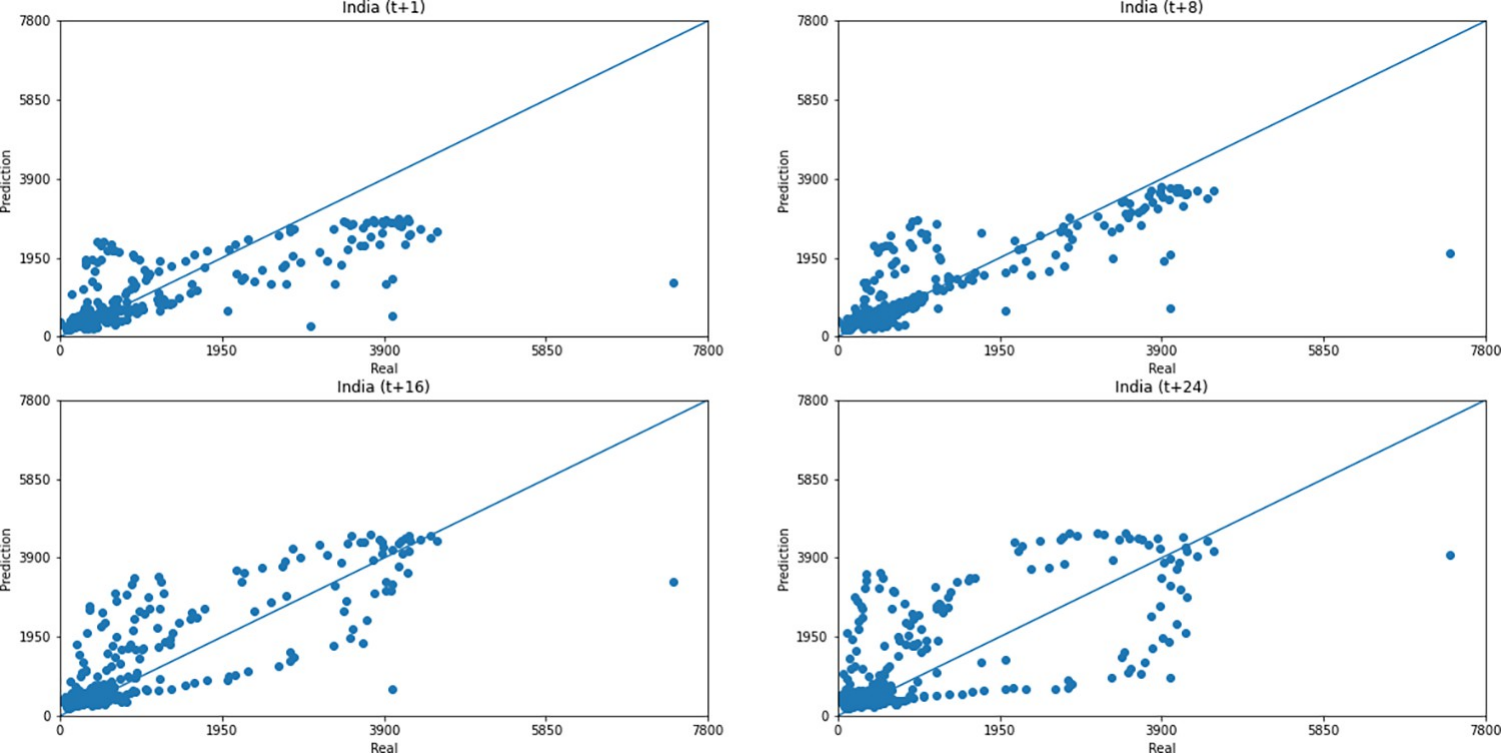
Scatter plot—predicted values vs. actual values for confirmed deaths–India.

**Fig 32:**
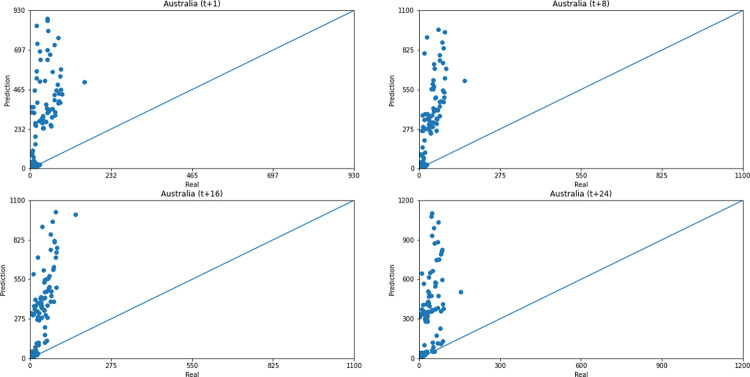
Scatter plot—predicted values vs. actual values for confirmed deaths–Australia.

**Fig 33:**
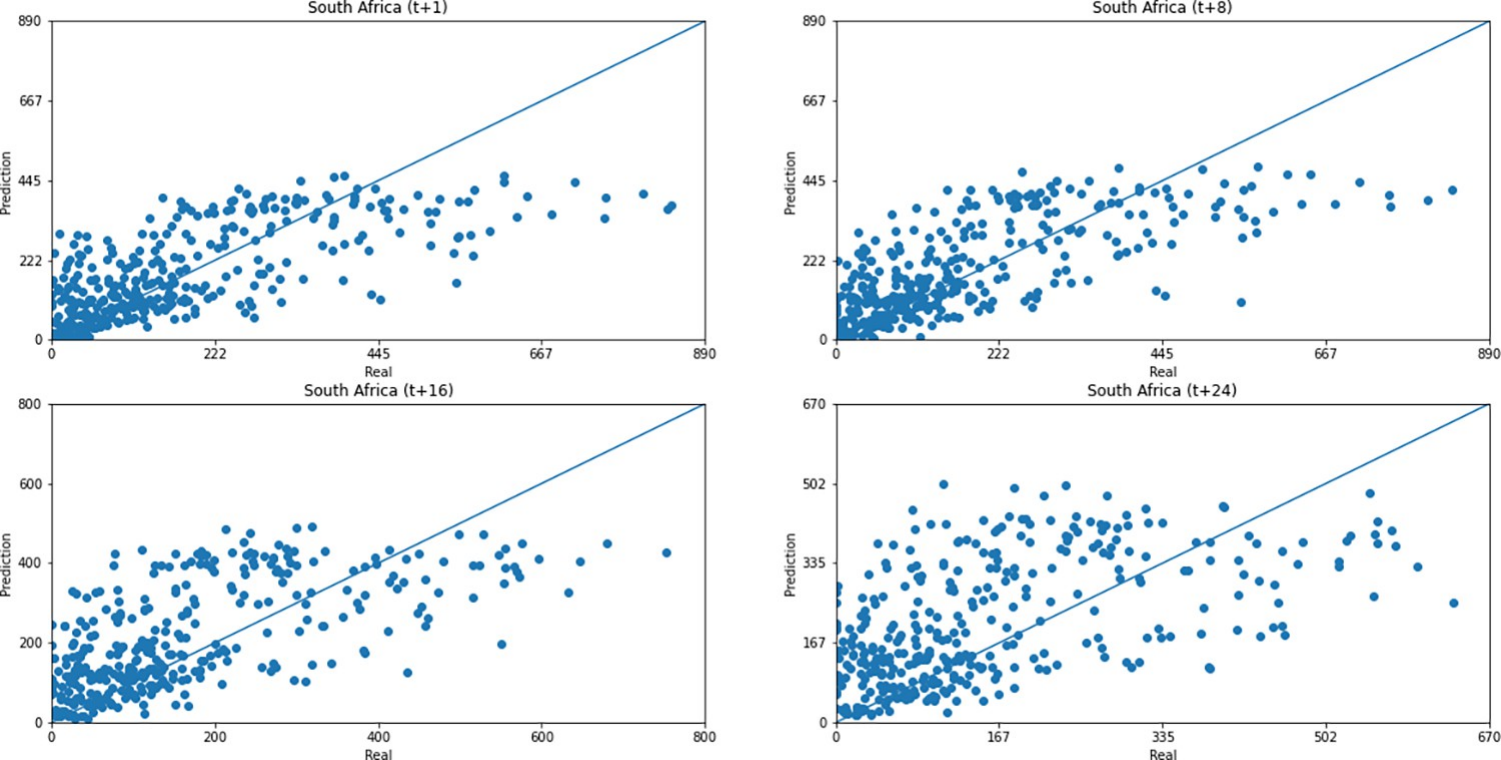
Scatter plot—predicted values vs. actual values for confirmed deaths–South Africa.

**Fig 34:**
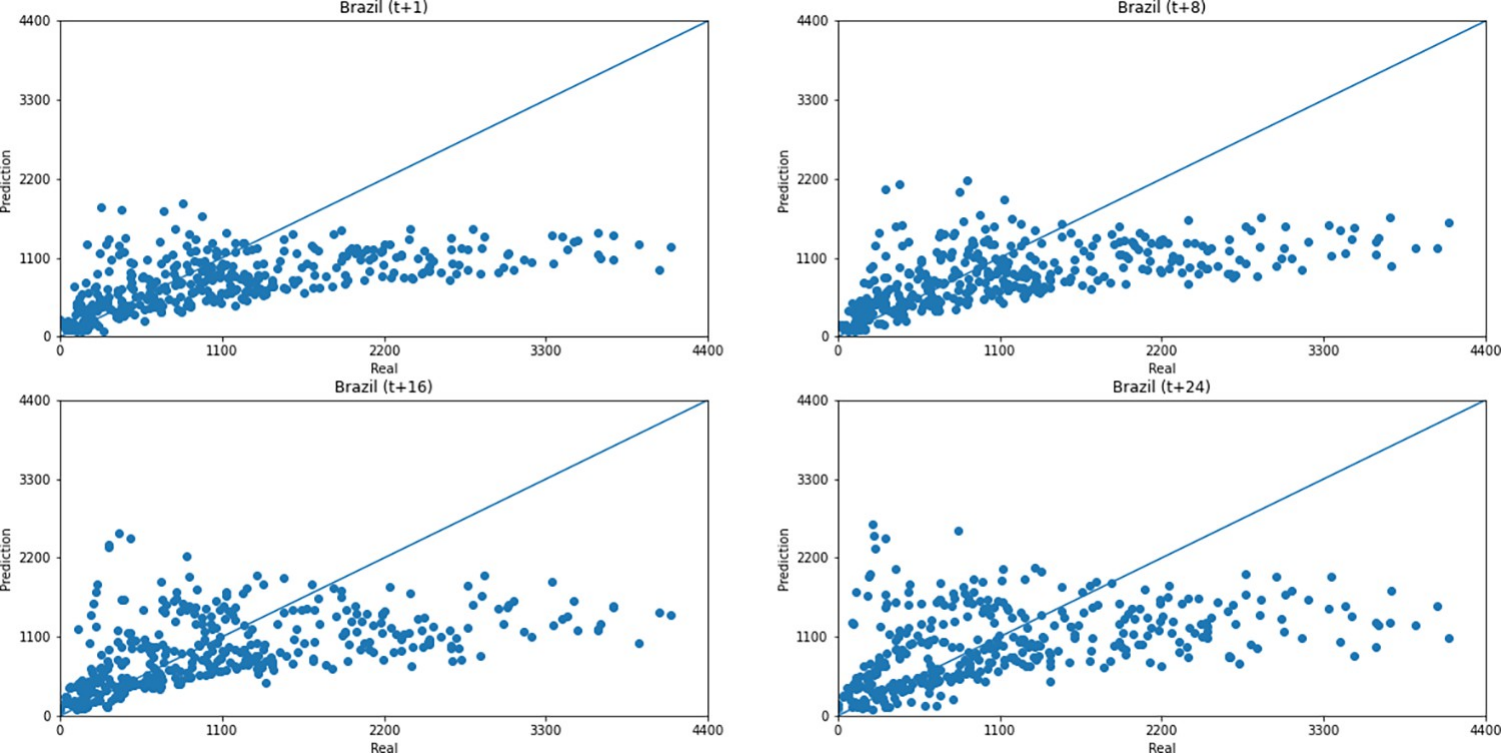
Scatter plot—predicted values vs. actual values for confirmed deaths–Brazil.

**Fig 35:**
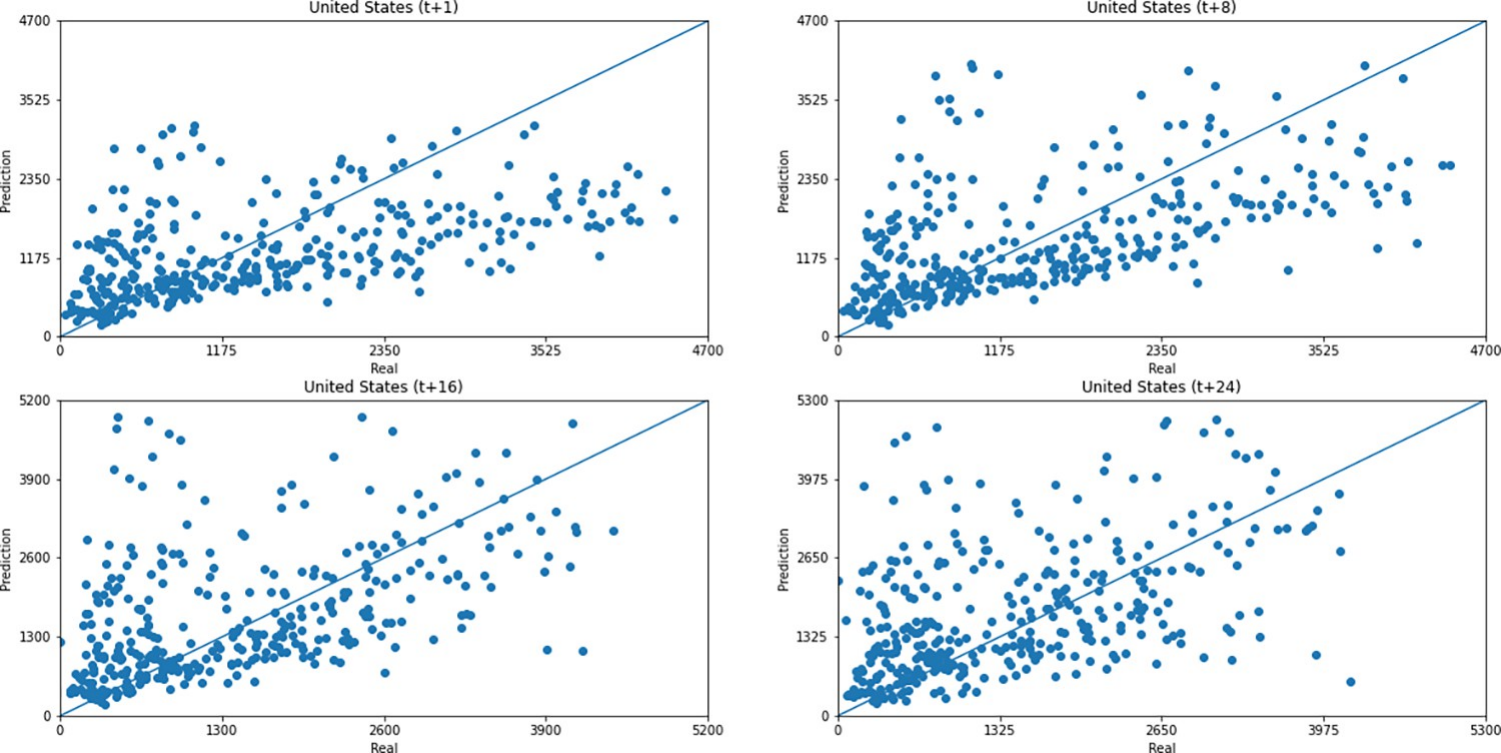
Scatter plot—predicted values vs. actual values for confirmed deaths–United States.

Concerning data dispersion, none of the countries presented a clear linear behavior in the scatter diagram, although India presents the highest Pearson R value in a prediction horizon (0.8715). Figs 30–35 present scatter plots from predicted and real deaths data for each of the six countries under analysis, considering four time-steps (t+1, t+8, t+16, t+24).

In general, the use of the same architecture to train a model to predict confirmed cases and deaths from COVID-19 showed satisfactory results. The hybrid model DWT+CNN+LSTM presented the best performance when compared to the other models tested for confirmed cases prediction. The same did not happen with deaths prediction: the qualitative evaluation of the model for predicting deaths showed some distance between the time-series constructed with the predicted values and the real series, mainly for the United Kingdom and Australia.

**Fig 36:**
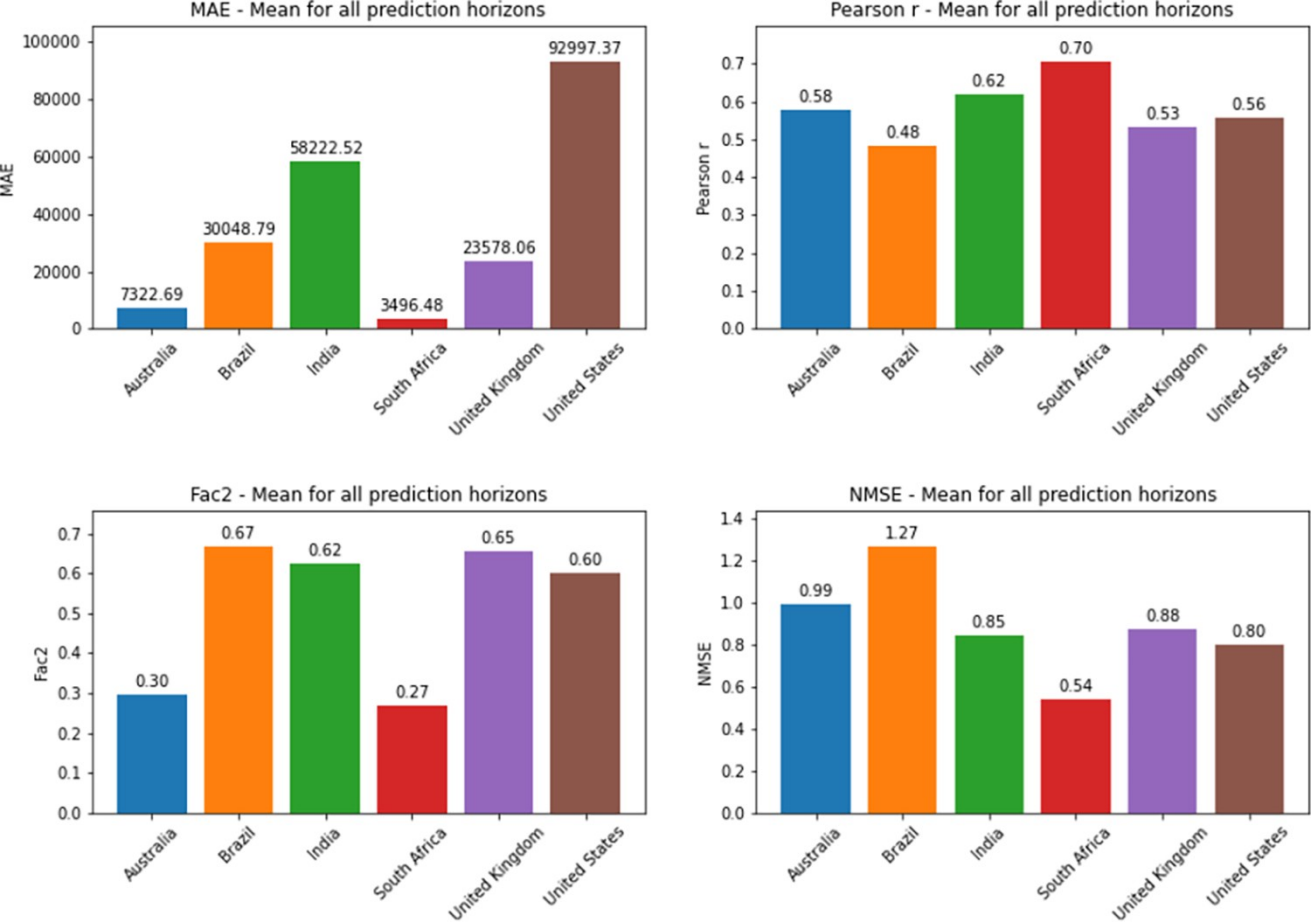
Average metrics from all prediction horizons for confirmed cases by country.

**Fig 37:**
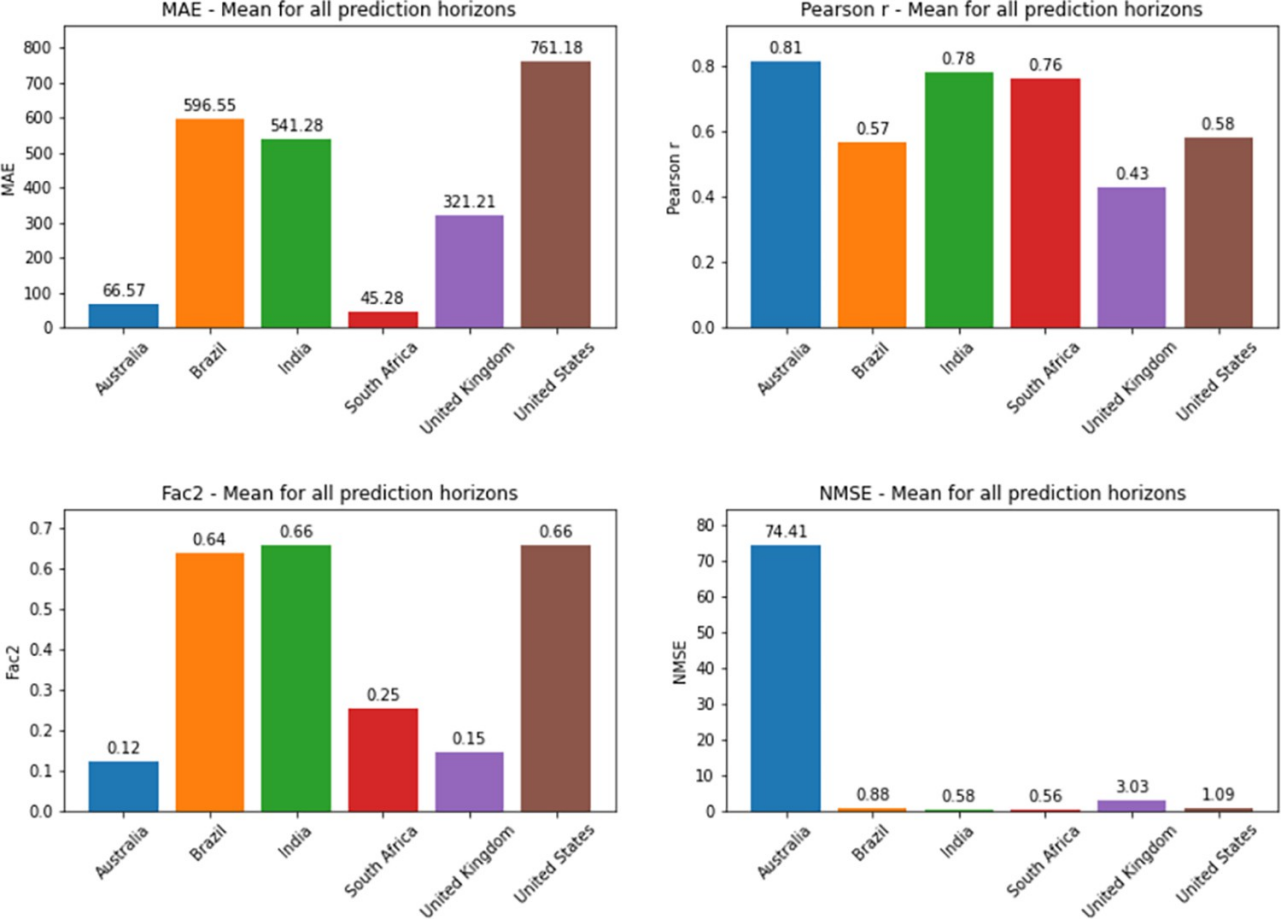
Average metrics from all prediction horizons for deaths by country.

Considering the average metrics from all prediction horizons in each country, Australia was the country which presented the worst NMSE values for deaths prediction. South Africa was the country that presented the best NMSE value for both confirmed cases and death series. For comparing different models or different implementations of the same model, NMSE is a good metric as it normalizes mean squared error (MSE), which facilitates the comparison of datasets with different scales. In this case, lower values indicate superior model performances. Detailed information on the comparison between countries is found in Figs 36 and 37.

As can be seen from the graphs in relation to the other metrics, the best Pearson R and MAE values for confirmed cases were also presented by the predictions for South Africa. The best average for Factor of 2 was presented by Brazil. For deaths prediction, South Africa presented the best MAE as well. The best Pearson R and Factor of 2 averages for deaths were presented by Australia and the United States, respectively (Factor of 2 values shown in the graph are approximations of 0.6589 for United States and 0.6566 for India).

Overall, South Africa showed good predictions for both confirmed cases and deaths, considering an evaluation of the DWT+CNN+LSTM model performance. This result is also corroborated by the qualitative analysis of the predictions (see Figs 15 and 27). It is worth remembering that the interval used for testing the model encompasses the same period in which the first cases of Omicron, variant of the virus that causes COVID-19 (SARS-CoV-2), were observed. The first cases in the world were documented in November 2021 by South Africa.

## 5. Conclusion

This paper presents a proposal for deep learning optimization modeling to predict the daily number of cases and deaths from COVID-19 worldwide data, using Wavelet decomposition. The model predicts the pandemic behavior for 30 days ahead, considering all countries in the world with available public data of confirmed cases and deaths. Promising results for the CNN+LSTM hybrid approach were found, which was potentiated with the application of Wavelet signals.

According to the results of our experiments, it can be observed that: (1) convolutional layers added to LSTM networks can make the model more accurate, since CNN allows the interpretation of data from different perspectives beyond the time series lags itself, which provides more information to learn; (2) for confirmed cases prediction, the usage of discrete Wavelet signals improved the performance CNN+LSTM; (3) in general, all tested models for deaths series demonstrated similar metrics to confirmed cases models, although the use of Wavelets did not substantially improve the models for deaths series; (4) the qualitative evaluation of the hybrid model DWT+CNN+LSTM for six countries showed satisfactory performance for both confirmed cases and deaths, with better results for the prediction of confirmed cases.

In addition, it is important to highlight that the model with Wavelet implementation was trained with data from September/2020 to December/2020. Although the amount of input variables is greater due to the use of the detailing and approximation coefficient signals of Wavelets at four levels, the time series used for training was smaller than the series used for the model without Wavelets, which used the complete series of available data in 2020. As seen, the hybrid model with Wavelets presented the best performance for the confirmed cases series. Although the same did not occur for the prediction of the deaths number, a satisfactory result was observed when the DWT+CNN+LSTM model was evaluated. Considering the size of the series used for training the models with Wavelets compared to models trained without Wavelets, the potential for optimizing the models with Wavelets application as a feature augmentation technique is evident.

It was also noticeable that our model was able to identify the rise of a new wave of confirmed and death cases, showing its potential to predict the pandemic behavior, including new infection waves from different variants of SARS-CoV-2.

Our suggestion for future work on this research is to insert new input variables, such as the contingency measures established in each region, population size, vaccinated people, among other variables that may affect public health issues, correlating and predicting the impact of contingency measures on the pandemic and the economy, as well as evaluating the relationship of these variables with the pandemic behavior on the developed models.

We believe this is an innovative study as it proposes a unique model trained with real data from all countries in the world, focusing a challenging middle range prediction horizon of 30 days ahead, in addition to using up-to-date techniques such as the use of feature augmentation based on Discrete Wavelets Transform. In addition to integrating information of all the diversity observed in the pandemic scenario between different countries, the study evaluates the possibility of using a single model that offers good predictive performance for any location in the world, both for the prediction of confirmed cases and deaths.
